# Making human pancreatic islet organoids: Progresses on the cell origins, biomaterials and three-dimensional technologies

**DOI:** 10.7150/thno.66670

**Published:** 2022-01-03

**Authors:** Lai Jiang, Yiru Shen, Yajing Liu, Lei Zhang, Wei Jiang

**Affiliations:** 1 Department of Biological Repositories, Frontier Science Center for Immunology and Metabolism, Medical Research Institute, Zhongnan Hospital of Wuhan University, Wuhan University, Wuhan 430071, China; 2 College of Life Sciences, Wuhan University, Wuhan 430071, China; 3 Asia Regenerative Medicine Ltd., Shenzhen 518110, China; 4 Human Genetics Resource Preservation Center of Wuhan University, Wuhan 430071, China; 5 Hubei Provincial Key Laboratory of Developmentally Originated Disease, Wuhan 430071, China

**Keywords:** pancreatic islet organoids, stem cells, diabetes, regenerative medicine, biomaterials

## Abstract

Diabetes is one of the most socially challenging health concerns. Even though islet transplantation has shown promise for insulin-dependent diabetes, there is still no effective method for curing diabetes due to the severe shortage of transplantable donors. In recent years, organoid technology has attracted lots of attention as organoid can mirror the human organ *in vivo* to the maximum extent *in vitro,* thus bridging the gap between cellular- and tissue/organ-level biological models. Concurrently, human pancreatic islet organoids are expected to be a considerable source of islet transplantation. To construct human islet-like organoids, the seeding cells, biomaterials and three-dimensional structure are three key elements. Herein, this review summarizes current progresses about the cell origins, biomaterials and advanced technology being applied to make human islet organoids, and discusses the advantages, shortcomings, and future challenges of them as well. We hope this review can offer a cross-disciplinary perspective to build human islet organoids and provide insights for tissue engineering and regenerative medicine.

## Introduction

Human pancreas is a composite organ that comprises acinar cells secreting digestive fluid, a duct system by which the fluid drains into the duodenum and the endocrine portion embedded in the exocrine parenchyma of the pancreas called islet (islet of Langerhans). Several types of endocrine cells are present in a human islet, 50% of which are insulin-secreting β cells, followed by glucagon-releasing α cells (35-40%), somatostatin-releasing δ cells (10-15%), pancreatic polypeptide-secreting PP cells and ghrelin-secreting ε cells 1. By secreting hormones, islets are primarily responsible for controlling glucose homeostasis, while the ablation or functional loss of β cells will lead to diabetes mellitus, a metabolic disease characterized by hyperglycemia 2.

There are around 425 million diabetic patients in the world, and the global incidence rate will increase to 552 million by 2030 as predicted 3. The current standard treatment for diabetic patients is administration of human recombinant insulin via multiple daily injections or insulin pump devices, which are basal-bolus therapies including a long-acting insulin that provides basal insulin and a rapid-acting insulin administered before meals 4. Although efforts have been underway to form a timing closed-loop system by combining insulin pumps with continuous glucose monitors, under a computer algorithm controlling, it still fails to recreate physiologic euglycemia. Hypoglycemia occurs in 31-41% of diabetic patients and this risk escalates with intensive insulin therapy and improved control of hyperglycemia 5. Therefore, the transplantation of isolated pancreatic islets becomes an alternative for diabetes treatment. In 2000, seven patients achieved insulin independence for 1 year by receiving around 800,000 pancreatic islets transplantation into the hepatic portal vein, and this procedure is universally known as the “Edmonton protocol” 6. In 2020, the National Institutes of Health-sponsored Clinical Islet Transplantation Consortium reported a phase 3 pivotal trial of human pancreatic islets transplantation in patients with type 1 diabetes (T1D). In this study, islet transplantation was effective with 62.5% of patients achieving the primary endpoint of freedom from severe hypoglycemic events and median HbA1c declined from 8.1% before to 6.0% at 1-year and 6.3% at 2- and 3-years after transplantation 7. However, islet transplantation is limited by a small donor pool and only available to the most brittle patients with unmanageable glycemic lability from the glucose monitoring, pump and/or intensive insulin injection therapy 5. Therefore, solving the problem of islet donor shortage is the most urgent challenge now.

Organoid, at very beginning, was used to describe organogenesis by cell dissociation and reaggregation in classic developmental biology experiments 8. Since the development of intestinal stem cell-derived organoid cultures by Clevers group in 2009, the definition of an organoid becomes a three-dimensional (3D) structure consisting of organ-specific cell types, which is derived from pluripotent stem cells (PSCs) or adult stem/progenitor cells 9, 10. However, the definition of an organoid has been nebulous, because many researchers have used vascular endothelial cells or adult cells which are not derived from PSCs to produce organoids recently. In brief, organoid is presented as “*in vitro* mini-organ” or somewhat “artificial organ”, which can mimic the *in vivo* organ landscape, exhibit an array of cell types found* in vivo* and demonstrate some aspects of the functions of the organ. Organoid technology has emerged as a tool to bridge the gap between cellular- and tissue/organ-level biological models, giving a more realistic representation of the *in vivo* tissue spatial organization and interactions between the cellular and extracellular environments, while retaining certain physiological functions 11.

Over the past decade, the islet organoid has attracted increasing attention as a promising model for diabetes to reveal the mechanism of islet-related diseases and an excellent platform to test the potency and toxicity of drugs. For instance, islet organoid can fill the blank of our knowledge about human pancreatic development between relevant early (<8 weeks gestation) and later gestational-aged human pancreatic tissue (>22 weeks gestation), and it is expected to be a new islet resource of transplantation in diabetes therapy 12-15. Many reviews have discussed the potential and significance of islet organoids, but none of them has given us a comprehensive summary of “how to make human pancreatic islet organoids”. Therefore, in this review, we summarize the cell types, biomaterials and technologies used to establish human islet organoids. We list the kinds of materials and methods, discuss their functions, advantages, challenges and outlook in the future. We hope to provide a method guide for making human islet organoids in a cross-disciplinary perspective and offer insights to tissue engineering **(Table 1)**.

## Cells for making human islet organoids

**Figure 1:**
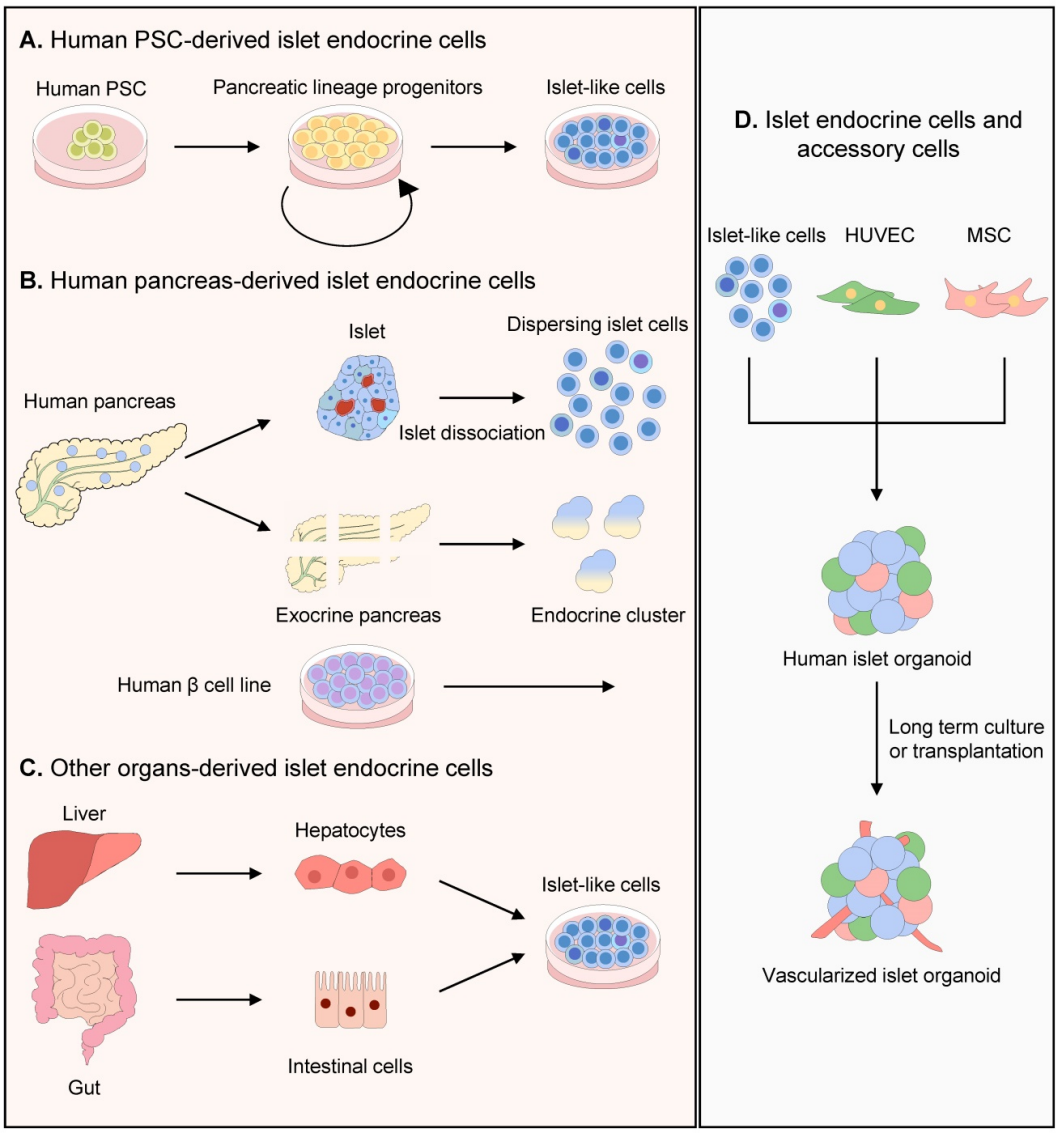
** Schematic illustration of cell origin for making human islet organoids.** (A-C) Many different strategies have been used to generate pancreatic endocrinal cell types, including islet lineage cells differentiated from human PSCs, islet cells and pancreatic progenitors derived from human pancreas, human β cell line and islet-like cells by trans-differentiation from non-islet lineage cells. (D) Accessory cells are proved to promote generation and vascularization of human islet organoids after long term culture or transplantation.

To construct islet-like functional organoids, pancreatic endocrinal cells as seeding cells are necessary to build up the organoid's main body. There are many different strategies to generate pancreatic endocrinal cell types appropriate for islet organoid culture, including 1) islet lineage cells derived from human PSCs; 2) islet endocrine cells which are enriched in human adult islets by dedifferentiation and redifferentiation; 3) non-islet lineage cells which can trans-differentiate into islet fate, such as duct, acinar cells and hepatic cells. The islet organoids with diverse seeding cells generated by different protocols exhibit significant discrepancy in structure and function. Meanwhile, some accessory cells have been proved to promote organoid function during islet-like organogenesis, including endothelial cells and mesenchymal cells **(Figure 1)**.

### PSC-derived islet lineage cells

Human PSCs, including embryonic stem cells (ESCs) and induced pluripotent stem cells (iPSCs), are considered desirable sources of islet lineage cells due to their virtually unlimited replicative capacity and the potential to produce almost all somatic cell types 65. Extensive works have been focused on generating β cells from PSCs and many achievements have been made in the last decades, which were summarized by several reviews 66-71. Therefore, many groups have utilized PSC-derived islet endocrine cells for making 3D human islet organoids. As early as 2001, human ESC line H9 was cultured in suspension to form the configuration of embryoid bodies. After 19 days of differentiation, these embryoid bodies had an average of 1-3% insulin-positive cells and more complex structures, such as epithelial- or endothelial-like cell structures or cysts 16. After that, pancreatic endocrine cells derived from human ESCs or iPSCs could be enriched by hand-picking or cultured lining hollow in hydrophobic plates to form cluster structures 17, 26, 72. Human ESC-oriented insulin-producing cells could also self-aggregate into insulin-positive cluster and be vascularized by transplantation or mixing with human umbilical vein endothelial cells (HUVECs) 25, 41. Besides differentiated endocrine cells from PSCs, the progenitor produced in process can also be a promising resource for islet organoid generation, which may considerably shorten the *in vitro* incubation time and reduce economic cost 73, 74.

### Islet endocrine cells from human islets or non-islet lineages

Dispersing human islet cells are also a promising cell resource for islet organoids 23, 33, 44. In these studies, human islets were dissociated into single cells and reaggregated into small islet organoids with standardized dimensions, which were also called pseudo-islets 23. The morphology of reaggregated human islets was similar to that of native islets and these pseudo-islets could improve diabetes reversal in diabetic mice 33, 44. Given the limited expansion capacity of matured islet endocrine cells, an attractive alternative could be the use of putative progenitor cells from pancreas that can give rise to the endocrine lineage 20, 38. In these studies, human islet organoids were generated successfully by either expanding adult pancreatic tissue or pancreatic progenitors digested from fetal pancreas 20, 38. Besides, endocrine progenitors derived from β cell dedifferentiation can differentiate to NGN3-negative, hormone-positive β (insulin)-, α (glucagon)- and δ (somatostatin)- “like” cells 75. Another study illustrated the construction of cell-to-cell and cell-matrix junction with a biomimetic scaffold to induce dedifferentiation of β-cells to undergo re-differentiation 76. Alternatively, trans-differentiation of endodermal origin differentiated cells has been applied in the generation of islet organoids as well, such as liver, gastrointestinal epithelium and exocrine pancreatic cells 24, 45, 77. However, the diverse culture efficiency of dedifferentiation and redifferentiation varies from case to case, and islet expansion is subject to much debate, which has restricted the development of islet organoids derived from human adult tissue. Therefore, efforts have been made for searching adult islet endocrine progenitors with capacities of expansion and differentiation into endocrine cells. Very recently, Wang and colleagues reported that a novel PROCR-positive endocrine progenitor found in adult mouse pancreas could generate new endocrine cells during adult homeostasis 78. These PROCR-positive progenitors can form functional islet organoids which are dominated by β cells and surrounded by a mantle of α, δ, and PP cells *in vitro*. However, it is still unknown whether human islets contain a counterpart progenitor population or not. Compared with PSC-derived endocrine cells, utilization of the cells from adult human tissues can avoid the risk of teratoma forming after transplantation, and pancreatic cells from diabetic patients can be a more natural source for human islet organoids generation.

### Accessory cells for islet organoids

Besides the islet endocrine cells, non-endocrine cells such as endothelial cells and mesenchymal cells also play a supportive role in islet survival and function. Pancreatic islets have a rich vascular supply, which offers sufficient oxygen, nutrient supply, fine-tuning blood glucose sensing and regulating of human islets. Endothelial cells which constitute vascular network can not only actively regulate vascular permeability, promote vascularization of islet organoids but also tend to behave as “guardians” to control the expression and movement of some important immune mediators 79. Moreover, endothelial cells can produce multiple factors, such as connective tissue growth factor, bone morphogenetic protein 2 (BMP-2) and BMP-4, which could increase the expression of insulin in adherent cultures of human ESC-derived pancreatic progenitor cells, and mediate islet-specific maturation 80-82. For example, HUVECs or endothelial cells have been mixed with human pancreatic progenitors or EndoC-βH1 β-cells for promoting islet organoids' self-clustering, maturation and vascularization 35, 39, 57. Besides the exogenous addition of endothelial cells, endothelial growth factors can induce the angiogenesis of human islet organoids 59. Recently, angiopoietin-2 has been proven to induce a wide spread of VE-cadherin^+^ endothelial cells and NG2^+^ pericytes within islet organoids. Moreover, angiopoietin-2 can partially supplement the* in vivo* paracrine stimulation from endothelial cells to β cells, which has empowered the human islet organoids with a native like glucose stimulated insulin secretion level by facilitating F-actin remodeling and regulating dynamics of calcium ion influx 59.

Similar to endothelial cells, mesenchymal cells were also found to take part in the construction of islet-like organoids, including self-organization, constitution of vascular network and paracrine function: (a) mesenchymal cells trigger the initiation of self-condensation, and this traction force produced by actomyosin cytoskeletal axis results in the directed and drastic movements of cell collectives 83, 84. In 2018, Takahashi and colleagues constructed self-organized islet-like organoids by co-culturing endocrinal MIN6 cells with HUVECs and mesenchymal stem cells (MSCs), which could normalize body weight and blood glucose levels of diabetic mice after transplantation 85. In the following research, human ESC-oriented pancreatic progenitors, MSCs and endothelial cells were seeded on Matrigel in an optimized cell ratio to self-organize the islet organoids 46; (b) In addition to triggering self-condensation, the mesenchymal cells supported endothelial cell interactions in an intramuscular human islet transplantation model: human islet endothelial cells migrated from the graft center into the surrounding tissue forming chimeric blood vessels with recipient endothelial cells 86; (c) Rat mesenchymal cells derived from bone marrow were reported to support encapsulated mouse islets within a hydrogel environment by the release of the trophic factors such as HGF and TGF-beta, and elevated insulin secretion appeared upon glucose challenge 87. For more information, another review summarized the many other kinds of accessory cells being used to co-culture with islet cells 88.

## Materials application of islet organoids

**Figure 2:**
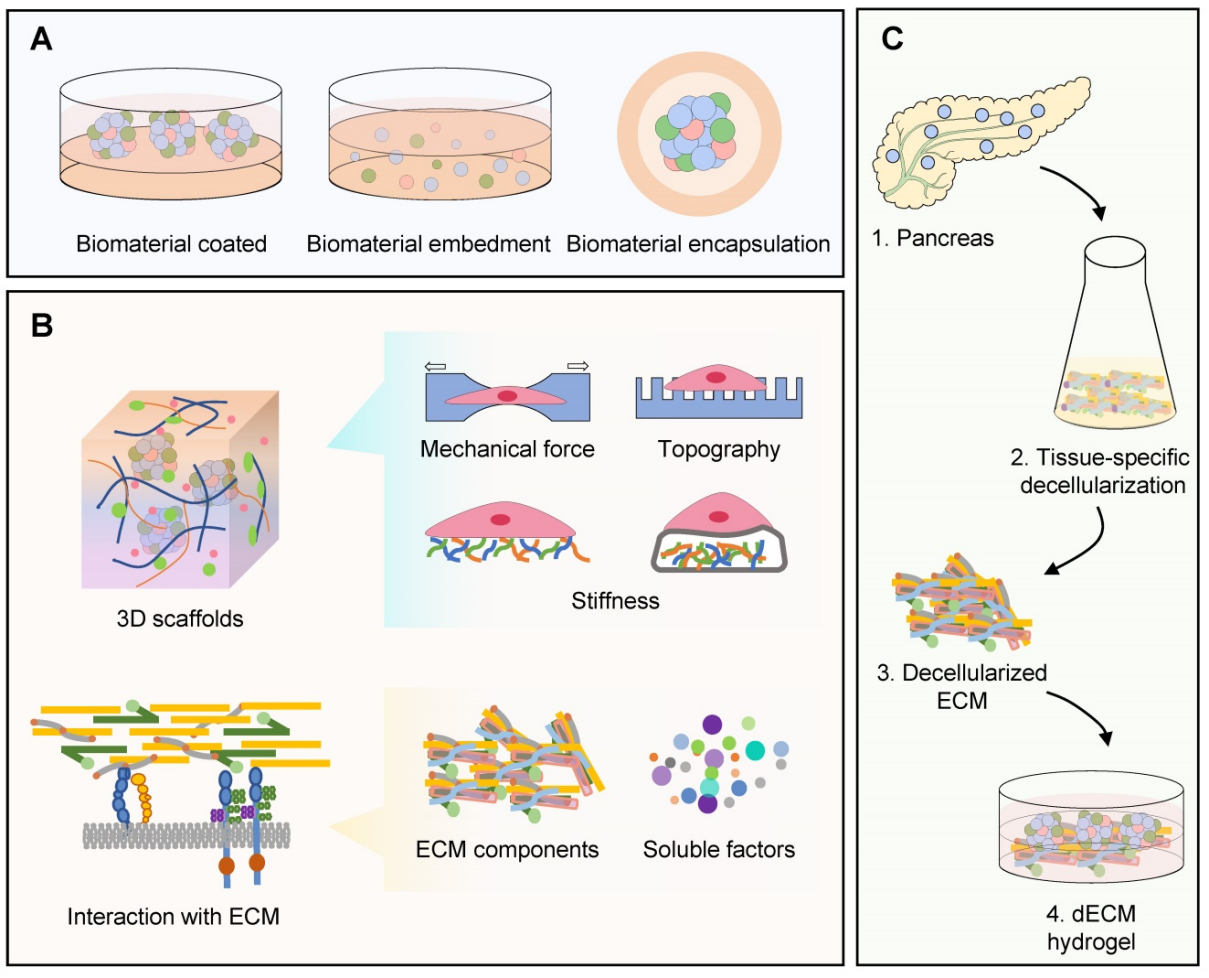
** Schematic illustration of materials application of human islet organoids.** (A) Usages of materials for producing human islet organoids, such as biomaterial coated, embedment and encapsulation. (B) Biomaterials can provide 3D scaffolds and mimic the native interaction with ECM for islet organoids generation, including mechanical force, topography, stiffness, signaling from ECM components and soluble factors. (C) Manufacturing process of dECM material.

Materials as one of the three predominant elements of tissue or organ 3D construction have attracted great attentions, and significant achievements have been made during the last decade. Materials serve as scaffolds with internal structures, ensuring the maintenance of the 3D structure of organoid. Their appropriate topological structure enables the formation of organoids in a controllable way. Moreover, materials used for 3D structure construction can usually provide a specific microenvironment for certain organogenesis *in vitro* 89. According to the materials' composition, they fall into natural and synthetic matrixes** (Figure 2)**.

### Natural matrixes used for human islet organoid construction

Natural matrixes are either extracellular matrix (ECM)-derived components or ECM analogues, whose chemical components or fibrous structures can mimic the native ECM of those tissues or organs *in vivo* 89. The cells can sense the physical, chemical and biological signals from ECMs, and accordingly alter a series of cell behavior such as migration, proliferation and matrix production 90. Cell-matrix interactions have been proven to improve β cell proliferation, insulin secretion and islet development 32. Based on the chemical nature, these natural matrixes are divided into three major groups: polysaccharide-based materials, protein/peptide-based materials (e.g., collagen, gelatin, elastin and fibrin), and decellularized ECM-based materials 90.

Polysaccharide-based materials are ideal for fabricating complex 3D scaffolds *in vitro* due to fast gelation properties 89, 90. Among them, alginate was applied on islet microencapsulation at first for the bioartificial endocrine pancreas in 1980 91. Apart from high biocompatibility for cell survival, alginate could protect islet cells from inflammatory response after transplantation due to its negative charge 92. Alginate has also been used for human islet organoids generation by encapsulating pancreatic endocrine progenitor cells derived from PSCs, because of the fast cross-linking of sodium alginate with calcium/barium chloride, and promotion to maturation of islet organoids by integrin 52, 58. However, alginate, which possesses low cell adhesion and cell interaction ability, fails to provide the cell anchorage required for cell survival 93. To overcome this defect, some bioactive substances are added into alginate matrixes, such as hyaluronic acid (HA) 94. HA is a major component of natural ECM synthesized by various types of islet endocrine cells, and usually locates in peri-islet. Alginate matrixes with the addition of HA were proved to promote the differentiation of MSC to insulin-producing cells and enhance insulin releasing 95. In 2020, a binary capsule relied on interfacial complexation of oppositely charged Na-alginate and chitosan was used to generate human islet organoids, in an all-in-water microfluidic system 53. This capsule exhibited high uniformity and provided 3D culture for human islet organoids in a continuous process by encapsulating pancreatic endocrine cells from iPSCs. With the excellent biological activity, safety and biodegradability, chitin is also a promising candidate for 3D culture. For example, macroporous chitin microspheres were proved to support the growth and spontaneous differentiation of human ESCs 96. Taken together, polysaccharide-based materials show great potentials for supporting 3D scaffolds and mimicking ECM for human islet organoid generation *in vitro*.

Protein/peptide-based materials are produced with matrix proteins existing in natural ECM, including collagen, gelatin, laminin, fibronectin and elastin. Among these proteins, collagen is the most diverse and abundant structural protein, and has been widely used for 3D stem cell cultures. In addition to building the artificial 3D structure for islet organoids, protein-based materials would also enhance islet organoids' function and maturity 32, 97. For example, high expression of Collagen I and IV was demonstrated to facilitate the development of human pancreas, and the combination of Collagen I and IV would lead to a higher insulin gene expression compared to fibronectin or laminin in the cultured human islets 98, 99. Proteomic analysis showed that collagens were proportionally higher in decellularized rat pancreatic extracellular matrix hydrogel compared to that in Matrigel (commonly used as supportive substrate for organoid culture) 51. The recapitulation and glucose-responsive hormone production of human islet organoids were promoted by the presence of Collagen V substrates 51. Recently, laminin, nidogen and collagen IV proteins were coated on the surfaces of human islet organoids to generate a basement membranes-like structure, which can enhance insulin secretion of β cells 60. Nevertheless, protein-based materials might introduce cell-dedifferentiation and protein alone is insufficient to replicate the natural ECM environment, which would hinder the further application in islet organoid construction 98.

Decellularized ECM (dECM)-based materials are obtained from decellularized tissues or organs which render the bioactivity and biocompatibility of its natural origin 90. Over the past few years, dECM-based materials have been extensively investigated. Compared with polysaccharide- or protein/peptide-based materials, dECM-based materials can mimic the niche of *in vivo* tissues or organs better 43, 100. In addition, *in vivo* ECMs have tissue-specific composition, including specific growth factors and macromolecular components, and mechanical properties. Therefore, dECM-based material can not only be a mere scaffold, but also be used to determine specific cell fate *in vitro* culture 101. The decellularized pancreatic ECM-based material becomes a suitable substrate for improving the microenvironment of islet cells and promotes the differentiation of human PSCs into pancreatic islet cells 102-104. The dECM materials made from rat or porcine pancreas have been used for islet organoids generation, and they are proved to promote human PSC-oriented islet cells differentiation and 3D islet-like organogenesis significantly 43, 50. Compared to traditional commercial Matrigel, cells differentiated from PSCs on pancreatic dECM material exhibited similar cellular composition with native pancreatic islets and presented increased islet signature markers expression and more insulin secretion in response to glucose stimulation. Collectively, dECM-based materials offer a nearly natural microenvironment for cells, yet there are several limitations that prevent them from the clinical application: (a) the process to obtain dECM gels, including decellularization, extraction, and purification, is time-consuming and costly; (b) batch-to-batch variability of raw material resources, especially the biomass material; (c) it is almost impossible to control biophysical properties and biochemical components; (d) these gels are derived from organisms that may suffer from cellular compatibility and pathogen transmission. For these reasons, the current dilemma of natural materials has been prompted the investigation of synthetic matrices to support organoid culture *in vitro* **(Table 2)** 105.

### Synthetic matrix material used for human islet organoids construction

Synthetic matrix materials applied in medication are generally biocompatible and non-toxic, with excellent characteristics such as controllability, reproducibility, degradation and good mechanical properties. The common synthetic polymers in previous reports include polyethylene glycol (PEG) and its derivates, polylactic acid (PLA), poly (lactic-co-glycolic acid) (PLGA), poly (vinyl alcohol) (PVA), poly(ε-caprolactone) (PCL) 89. According to specific applications, synthetic matrix can be customized to different characteristics, for instance, providing ECM characteristics for enhancing cell-cell interaction* in vitro* and mimicking stiffness of native islet, which makes synthetic matrixes as a promising substrate material 93, 106. In the previous research, several studies indicated the high potential application of synthetic matrix in tissue engineering, such as 3D printed PLA trapper device for human islet organoids transplantation, PVA or PCL/PVA 3D scaffold for β cells differentiation and islet organization 34, 36, 46. PCL is a kind of biocompatible and biodegradable polyester with high mechanical stability, and its poor cell affinity can be improved by mixing PCL with PVA which is affordable, highly thermal stable and hydrophilic. In these studies, the PVA or PCL/PVA nanofiber scaffolds enhanced the differentiation and functionality of β cells, producing human islet-like organoids similar to spherical shaped islets of Langerhans 34, 36. In addition, synthetic matrixes like PLGA and PLA were successfully utilized in islets transplantation and islet organoids production 107-109. PEG-based matrix is also a versatile tool for modeling cellular microenvironments and providing platform for human islet organoids 21. Compared to suspension culture, the 3D culture platform made from PEG/Collagen I mix matrix has advantages for human islet organoids: (a) enabling long-term culture of islet organoids, (b) maintaining aggregate size and morphology, (c) not adversely affecting islet differentiation and (d) providing a means for aggregate recovery 21. To improve the low cell-matrix interaction of synthetic matrix, scientists mixed natural components with synthetic polymers, such as gelatin/PLGA and PEG/Collagen I matrix, which are also defined as semi-synthetic matrix materials 21, 108.

## Approach for producing human islet-like organoid

**Figure 3:**
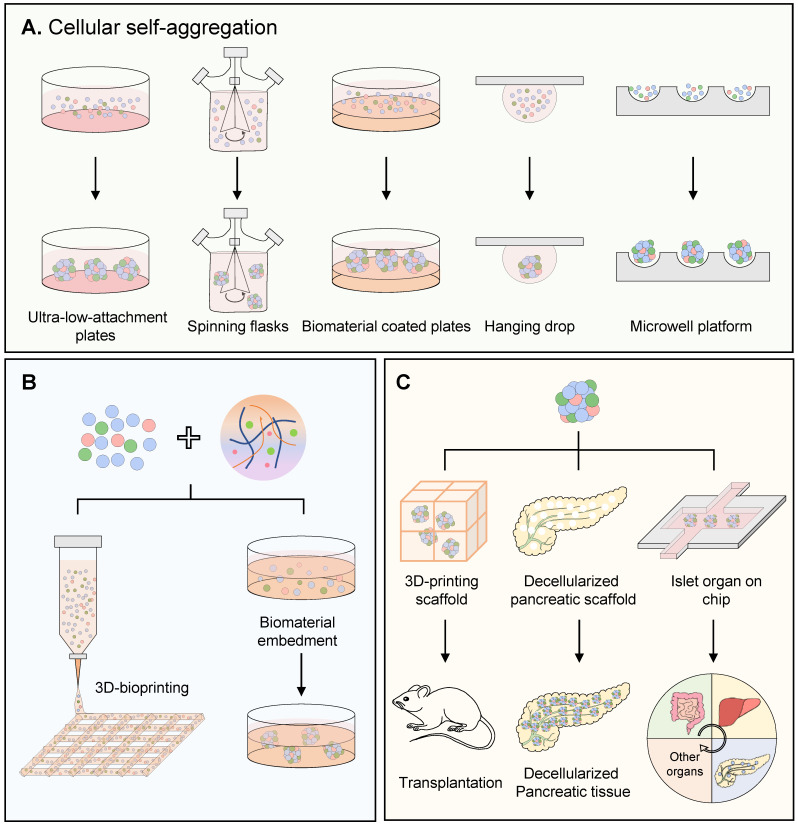
** Schematic illustration of approaches for producing human islet-like organoids.** (A) Methods of constructing islet organoid 3D structure by cellular self-aggregation, including seeding cells on ultra-low-attachment plates, in spinning flasks, on biomaterial coated plates, by hanging drop and in microwell platform. (B) Mixing human islet lineage cells with hydrogel and generating 3D structure by 3D bioprinting or biomaterial embedment. (C) Methods of maintaining human islet organoids, including basketed in 3D-printed scaffold; seeded into decellularized pancreatic scaffold to form native like structure; cultured on a chip which makes it possible to model inter-organ communication in diabetes pathogenesis.

Compared with conventional monolayer culture, 3D provides islet organoid a more realistic environment and structural organization similar to native human islet 110. The standard methods of constructing islet organoid 3D structure consist of traditional self-aggregation, modified self-aggregation and hydrogel-based embedment. The newly emerged methods such as 3D bioprinting, decellularized organ scaffold and organ-on-a-chip will be discussed as well** (Figure 3)**.

### Traditional cellular self-aggregation for islet organoid's 3D structure

Cellular self-aggregation (self-organization), widely demonstrated in developmental biology, is a process that dissociated cells could self-assemble into tissue-like structures 111. Additional factors, including low adherent culture material, accessory cells and soft substrate, can promote cellular self-aggregation. For example, low-attached plates can stimulate the human PSC-derived dissociated pancreatic progenitors, endocrine cells or human β cells to cluster into 3D islet-like organoids spontaneously 18, 27, 28. Spinning flasks providing suspension culture conditions can induce the 3D structure for islet organoids 15, 48. Human ESCs were suspended to form embryoid bodies in spinning flasks to gain 3D structure, followed by a stepwise islet endocrine differentiation 15. In addition, the mesenchymal cells could assist the initiation of self-aggregation and improve the aggregate function 85. The MSCs triggered self-organized islet-like organoid was placed in a 3D‐printed tissue trapper and implanted into mice for 90 days, with detective vessels, a higher number of insulin-positive cells and human C-peptide secretions 46. The soft substrate could also help the cellular self-aggregation. In 2018, a novel engineered hydrogel system named Amikagel, was established for regenerative islet organoids 35. Amikagel-coated plate facilitated spontaneous aggregation of human pancreatic progenitor cells derived from ESCs into robust homogeneous spheroids, which resulted in significant enhancement of islet phenotype compared to the widely used Matrigel. Additionally, other materials are proven to promote the self-aggregation in islet organoid formation, such as silk matrices made of recombinant spider silk protein, albeit there was no clear explanation about the silk matrices' function in self-organization process of human islet cells 29. Thus, the traditional *in vitro* cellular self-aggregation could generate impressive microscopic and functional complexity of organoids. However, lacking in pre-defined extrinsic patterning instructions, traditional cellular self-aggregation always leads to a heterogeneous organoid population with a large variety in aggregate dimensions, and the number of cells per aggregate was considered to play a role in cell cluster differentiation and functionality 112.

### Modified cellular self-aggregation for islet organoid's 3D structure

Determining the size of cluster is important for human islet organoids generation. Large size of islet would induce an increased necrosis and decreased cell viability compared to small size islets, no matter *in vitro* culture or implantation 113. The cellular insulin content of small size human islet organoids (250 cells) is up to 2.8-fold higher than their large counterparts (1500 cells) 33. To obtain the homo-sized organoids, alternative methods are accordingly developed for somewhat controlled cell self-aggregation, such as hanging drop, microcontact printing and microwell platforms. The hanging drop procedure forming cell clusters by gravity, is generally used for embryoid body formation, and completely out of contact with any artificial supporting matrices or surfaces. Montanari and colleagues reported the generation of islet organoids by mixing single human islet cells with mesenchymal cells in the hanging microdroplets, which had functional insulin secretion *in vivo* 31. By hanging drop, the size of islet organoids can be adjusted by varying concentration of cell suspension, droplet volume and incubation time. However, this method is labor-intensive and not applicable to large-scale engineering 23. Mendelsohn and colleagues reported a microcontact printing strategy by printing spots of the cell-adhesive protein-laminin covalently linked on glass coverslips 114. This technique offers exceptional control of cell clusters' shape and size, and the high-speed printing makes a high-throughput manner to produce β cell aggregates. However, these cell clusters have only two to three cell layers and even could not be removed from the substrates. Thus, they do not apply to pancreatic islet models, biological tests or implantation either. At present, microwell may be a promising method for self-organization-based islet organoids generation 115. Microwell is comprised of multiple micro-meter-size compartments, generated by photolithography or micropatterning, with an extensive range of materials, such as polydimethylsiloxane (PDMS), PEG and agarose hydrogels 116. On agarose microwell platform, cells were trapped and aggregated into glucose-responsive “pseudo-islets” or insulin-producing organoids with stable and uniform size, which improved the survival of islet cell after transplantation 23, 40, 44. Moreover, the thinner bottom of concave microwell enhanced oxygen permeability for rat islet spheroids, because under the sufficient oxygen delivery conditions, islet spheroids presented long-term sustainability, enhanced viability, and increased hormone secretion 117. Thin film microwell array scaffolds coated with combinatorial ECM proteins were proved to promote human islet function and survival rate 118. However, limitations of microwell still exist, such as a narrow size range of cell aggregates, special procedures required for microwell preparation and low controllability of cell distribution 119. Moreover, although self-aggregated organoids with suspension displayed superb nutrient diffusion, the lack of certain physicochemical supports like natural organ niche would reduce the stability of islet organoid and cell viability.

### Hydrogel based embedment and encapsulation for human islet organoids

In addition to self-aggregation, embedding cells in hydrogel matrix is also a promising method to establish the 3D structure of islet organoids. In this method, isolated cell pellets are usually suspended in a polymer solution until cells are evenly distributed, followed with hydrogel formation by the polymer's crosslink. Hydrogel matrix provides an initial guidance to the aggregating cells and serves as a subsequent physical support and constraint for islet organoids formation. For example, islet organoids could be established by embedding human ESCs with mixture of collagen I and Matrigel solution to form a 3D scaffold that has an initial radius of 8 mm and a thickness of 2.5 mm 32. In another report, small pieces of human fetal pancreatic tissue were plated in Matrigel and expanded into organoid with islet endocrine fate 38. Recently, embedding pancreatic progenitors in alginate was reported to boost the islet-cell signature during the islet organoid formation process 52. Pancreatic progenitors derived from human iPSCs were embedded within the alginate beads by single cells suspension, which exhibited a highly significant increase in the proportion of insulin, glucagon and somatostatin expression compared with 2D culture. By global proteomics, Legoy and colleagues found that embedment with alginate promoted a large battery of proteins towards islet-like abundance levels, which made islet-like organoids more mature. Pathway analysis suggested that organoid with hydrogel embedment regulate the proteome landscape via integrins 52.

Islet organoids established by embedding cells in hydrogel not only can form 3D structure of islet cells, provide cell-ECM interactions which mimic the natural niche of islet, but also contributes to its long-term survival. To maintain the islet sustainability *in vivo* after transplantation, “islet encapsulation” may be a good strategy. The encapsulation method is like a capsule composed of islets surrounded by matrix materials 120. Microencapsulation of islets into alginate microbeads was first applied in the 1980s 91. The hydrogel-based encapsulation could reconstruct the native islet 3D environment and provide islet cells with a physical semi-permeable barrier that prevents infiltration of immune cells 121, 122. Fukuda and colleagues reported that islet-like spheroids derived from human iPSCs, which were encapsulated with alginate and followed with formation of fiber by conical tube, ameliorated hyperglycemia and maintained constant human C-eptide levels in the mice plasma 42. Moreover, these alginate fibers were retrieved easily with forceps without any adhesion. Besides, encapsulating islet organoids with hydrogel could also be long-term cultured *in vitro*. For instance, human islets were encapsulated with ECM hydrogel derived from porcine decellularized pancreas or the formulated spider silk protein, improving functional stability of human islet *in vitro* culture and mimicking native islet composition and morphology better 123, 124. Therefore, encapsulating islet organoids with matrix materials is also a promising method to promote the stability of islet organoids* in vitro* culture. Recently, Stock and colleagues encapsulated islet organoids derived from human stem cells with a conformal coating made of poly (ethylene glycol)-maleimide (PEG-MAL) crosslinked with dithiolated PEG (SH-PEG-SH), supplemented with PepGel peptide and PEG-oligoethylene sulfide (PEG-OES) nanofibrils 54. In this study, the islet-like organoids maintained a comparable insulin secretion profile and stimulation index *in vitro* with those of unencapsulated islets for up to 7 days, and even prevented the central necrosis which was visible in islets encapsulated in alginate and PEG-alginate larger microcapsules. Moreover, the islet organoids were transplanted into diabetic mice, which allowed long-term diabetes reversal and maintained euglycemia for more than 80 days without immunosuppression 54. Despite these achievements, there are still several limitations in this encapsulation application, for example, the implanted capsules cannot be fixed in a precise location and not be easily and completely removed from the patient due to the small size of the encapsulated islet; additionally, when the encapsulation is in a larger configuration, there is a diffusional problem that leads to poor diffusion of oxygen dramatically impairing the islets' viability; moreover, the lifespan and expansion of islets after encapsulation are still not good enough for the following medical applications.

### 3D bioprinting for human islet organoids

Building human organoids via 3D bioprinting technology has received particular attention in these years. By imitating the natural heterogeneous architectures of organ, organoid formation is expected to be capable of positioning different cell types in desired locations, or inducing progenitor cells into the desired type at specific locations, like printing 125. 3D bioprinting technology could print and pattern all the components into 3D structure with high precision and repeatability, including spatially distributed cells, biomaterials (bioink), and bioactive factors 126. Thus, 3D bioprinting provides an ideal strategy to construct organoid. Nowadays, 3D bioprinting has been widely explored for fabrication of liver, cartilage, neuronal tissue, osteochondral grafts and heart valve conduits 127. In the aspect of islets, PLA and fibrin hydrogel were used to house human PSC-derived β-cell clusters by 3D printing. Upon transplantation into mice, SC-β cell-embedded 3D-printed devices functioned for 12 weeks, were retrievable, and maintained structural integrity 109. Duin and colleagues produced macro-porous hydrogel constructs with embedded rat islets by 3D extrusion bioprinting 128. This macro-porous construct could increase the surface to volume ratios and reduce the distance between islets and the surrounding body fluid/blood vessels to less than a few hundred micrometers. Islets embedded in bioink (alginate and methylcellulose) with defined geometry were detected with viability values around 80% and glucose responsiveness for up to seven days in culture 128.

In addition, 3D bioprinting technology offers a unique role in the fabrication of islet tissue-like constructs with vascular structures, through its potential in recreating complex morphologies and multicellular environments 129. Recently, a coaxial configuration with mouse islets (core) and endothelial progenitor cells (protective shell), was produced based on 3D alginate-gelatin bioprinting 130. In this work, 3D bioprinting was demonstrated to precisely control over the distribution of multiple cell types, which had the potential to improve revascularization and suppress immune response. By making microchannels, 3D bioprinting mimicked natural vascular trees to perfuse islets with sufficient oxygen and nutrient supply. Additionally, bioinks may also incorporate endothelial cells plus slow-releasing compounds, such as VEGF, to promote angiogenesis surrounding the structure 121. dECM of pancreas is proved to be a promising bioink for islet organoids by 3D bioprinting, and dECM has shown shear thinning behavior, which is beneficial for the viability of the embedded cells as the shear stress was relieved when passing through the printing nozzle 43, 64, 126. Despite the revolutionary bioprinting technology approach and boundless potential for improvement in artificial pancreas fabrication, the early stages of its technical development barricade it from production preparation process. What required to be solved are (a) suitable bioink: The choice of bioink materials is limited by stringent printing conditions, almost all hydrogels are brittle and not stable enough to guarantee long-term survival of the islets. There are few available standards or commercial bioinks with good biocompatibility, appropriate biological and physicochemical properties; (b) printing speed: with the current bioprinting technology, human-scale tissues and organs would require too prolonged time for the printing process, thereby affecting the cell viability of the printed cells 131.

### Decellularized organ scaffolds for islet organoids

At present, islet or other organoids established *in vitro* suffer from size limitation, ranged in the millimeter scale, which restricts their direct usage in transplantation. To resolve this issue, decellularized scaffolds from human cadavers could be made and seeded with dissociated organ-specific precursors or directly with organoids containing parenchymal, vascular and other accessory cell types. Together with physiologically appropriate culturing methods and bioreactor cultures, these scaffolds could physically support the growth of larger organ structures *in vitro*. In 2013, decellularized mouse pancreatic scaffolds were generated by perfusion which could be recellularized with acinar and β cell lines, subsequently, cell-free pig pancreatic scaffolds were also seeded with porcine islets and human amniotic fluid-derived stem cells 132, 133. Thus, decellularized scaffolds showed a promising bioengineered platform to generate native-like artificial pancreas or islet. In the following studies, different perfusion routes (artery, portal vein, pancreatic duct) were compared and intact islets infused via the pancreatic duct was proved to be the viable and functional method 134. In another study, islets were engrafted in a decellularized rat liver, incubated with endothelial cells and mesenchymal stromal cells, which was proved to be an endothelialized neo-pancreas with function of insulin secretion 135. Despite the merits of decellularized scaffold, biomedical researchers now face the challenge of how to recellularize these organ scaffolds adequately and efficiently, especially in multiple cell type perfusion conditions.

### Human islet organ-on-a-chip

Organ-on-a-chip is a newly immerged conception. Incorporated with microfluidic, organoids gain significant advantages in offering biomimetic cellular or tissue microenvironments 136. Compared to static cultures, microfluidic introduces a perfusive flow facilitating transport of nutrients, oxygen and waste to mimic blood flow 137. An organ-on-a-chip microfluidic device had been developed to facilitate the formation of embryonic bodies from iPSCs and 3D islet organoids, which could improve the islet organoids' function and maturation 47. Recently, Walker and colleagues produced islet organoids with dispersing human islet cells by hanging drop, and cultured islet organoids in microfluidic system to promote their survival 56. In the following research, Oxy-Chip came up to support primary human islet organoids with better oxygen availability and islet function, in which the poly(methyl methacrylate) base of the chip well was replaced with an oxygen-permeable perfluoro alkoxy (PFA) membrane 138. Microfluidic platforms could also be used to create 3D models with different cell types maintained within separated compartments and connected by arrays of microchannels 139. A human microfluidic organ-chip platform was developed to study the connection among thymic epithelia, hematopoietic stem cells, islet β cells in T1D and pancreatic islet-liver cross-talking in type 2 diabetes (T2D) 140. Therefore, inter-organ communication in diabetes would be modelled by human microfluidic organ-chip platform 141, 142. Moreover, islet organoids with microfluidic systems could also be a simple, advanced method for high-throughput drug screening, which was proven in 2019 143, 144. The effect of slow flow dynamic conditions and small size of homogeneous reaggregated islet organoids made this microfluidic platform well-maintained for up to 1 month 144. As a result, islet organoids under dynamic conditions may exhibit higher sensitivity to drugs, such as tolbutamide and GLP-1, compared to static and conventional models (native islets with heterogeneity sizes). In conclusion, islet organoids combined with microfluidic systems will be a perfect platform for disease modeling, high-throughput drug analyses and regenerative medicine 145.

## Mechanical stimulation in the construction of human islet organoids

The effect of mechanical stimulation on cells has attracted much attention nowadays. It is reported that mechanical signals could influence the differentiation and function of β cells 146-148. In case of human islet organoids construction, mechanical stimulation also plays an important role. Geometry brought by cell aggregating is considered to be the most directly mechanical stimulation during islet organoids generation. Hogrebe and colleagues revealed that the state of cell cytoskeleton in flask 3D spinning culture is different from 2D adherent culture, which robustly affected β cells' directional differentiation 147. The presence of biomaterials would induce several mechanical stimulations to human islet organoids, such as stiffness, topography and mechanical pressure. Stiffness of biomaterials (Young's modulus) can regulate cell self-clustering for human islet organoids construction and β cell differentiation 104 35. Topography can be perceived by β cells and converted into biological signals which promote long-term human islet culture* in vitro* 149. Shalaly and colleagues reported the special topographical structure of silk foam matrices could promote human islet cells self-organization 29. Kim and colleagues found an optimal topographical structure for human islet organoids by using nano-scale polystyrene surface which increased the PDX1 expression during pancreatic differentiation of ESCs and promoted 3D cluster formation, when comparing with other nanopatterns or flat surfaces 49. Mechanical pressure given by biomaterials encapsulation is another mechanical stimulation. Legoy and colleagues claimed it could promote endocrine hormones secretion of human islet organoids via integrin signal 52. Shear stress is one of the most important mechanical stimulations in 3D bioprinting and microfluidic platform. In islet 3D bioprinting, it is essential to optimize the shear stress (pressure, nozzle diameter, printing speed), which can reduce cell viability by up to 40-85%. Klak and colleagues found that comparing with the large size islets, smaller size human islets (50-100 μm) have survival advantage under the same mechanical pressures, and the optimal pressure by the extrusion method should be lower than 30 kPa while using 3% (w/v) alginate as a carrier 150. In a microfluidic platform, the shear stress is determined by flow rate and affects the morphology and function of islet organoids 56. Dynamic condition with slower perfusion was detected to improve the abundant microvilli, tight cell junctions and enhanced glucose responsiveness of rat islet organoids, whereas in higher perfusion or static condition was the opposite 144.

In summary, cell aggregating, biomaterials and technologies used in the construction of human islet organoids all can influence the survival, differentiation and function of organoids by mechanical stimulations. At present, the underlying mechanism mainly focuses on the cell death caused by mechanical damage, regulating mechanical signals such as YAP, integrin and cytoskeleton dynamics. The optimal mechanical environment most suitable for human islet organoids has not been defined. In human pancreas, islets are surrounded with exocrine portion and abundant vascular tissue, which can provide stiffness, topography and mechanical pressure to islet. Therefore, the future research of human islet organoids' construction should not only focus on organoid itself, but also understand the native islets mechanical environment.

## Challenges and perspectives

In the past several years, single cell RNA-seq, CRISPR/Cas9, new biocompatible material, microenvironment induction and many other newly emerged techniques have been applied to screen the appropriate organoid development strategy, and exciting attempts and accomplishments have been made. Even so, some serious concerns of human islet organoids still exist.

### The gap between the islet organoid and native islet in terms of cell composition

(1) **Deficient cell types**. At present, most human islet organoids are only composed of β cells or endocrine progenitors. Few reports have attempted to design a proportion of α, β, δ and other accessory cells in making islet organoid (Table 1), which led to the different structures between islet organoid and native islet and may also lead to the immature status of β cells. There have been many robust differentiation approaches to generate β cells 151, 152, and these β cells somewhat still resemble the immature stage observed in the fetal pancreas 153. Most β cells differentiation approaches are based on the combination of growth factors and small chemical molecules targeting specific signal pathway, such as WNT 154-156, but the internal secretion regulatory and physical interactions surrounding the β cells were ignored 15, 157, 158. A few efforts have been made to generate α cells via directed differentiation or trans-differentiation, integrating into islet organoids 159, 160. Besides the endocrine cells, the known accessory cell types include but are not limited to endothelial cells, nerve fibers (neurocyte and neurogliocyte) and immune cells 161. The co-culture effects and methods of accessory cells with islet cells have been summarized 88. Nonetheless, most contents in this review were based on mouse islet cells, which was not sufficient to provide guidance for human islet organoids. Thus, a lot of hypotheses still need to be verified in making human islet organoids.

(2) **Different cell proportions.** Human islets consisted of a proportion of ~60% β cells and ~30% α cells 162, 163.* In vivo*, this proportion does vary with individuals in terms of age and islet size. The β- to α-cell ratio increased 6- to 7-fold from developmental preterm to childhood 164, and the β- to α-cell ratio in small size islets (defined as 40-60 μm in diameter) is nearly double of that in large size islets (> 300 μm in diameter) 165. To explore whether the proportion of cells will affect the function of islets, Kojima and colleagues aggregated the immortalized mouse pancreatic α and β cell lines into clusters, and found that the insulin secretion at a 1:8 α/β-cell ratio was higher than that of a 1:16 or 1:4 ratio 166. Therefore, checking out the cell proportion in human islet organoids generation is necessary. Hogrebe and colleagues reported that in human islet-like clusters, the majority of endocrine cells were detected to be β cells and the numbers of α and δ cells were much lower than those in human islet 62. Hence, adjusting the cell proportion may be an approach in improving functionality of human islet organoids. However, there are still many challenges to determine the most suitable cell proportions for the various cell types involved in islet development.

(3) **Disordered cell arrangement.** Most human islet organoid reported lacks the natural topological cell arrangement, extracellular matrix association and internal secretion microenvironment, despite it has much better performance than 2D culture. The cell arrangements of human islet organoids are different from human cadaveric islets. Human β cells were detected in the core of fetal islets and the small size human islets, surrounded by a mantle of α and δ cells 164, 165, 167. In large size human islets, β cells and α cells are intermingled in the islet core 167. In contrast, unlike native islets, human islet organoids showed a core shell configuration in which the mantle was comprised predominantly of β cells and the core was comprised predominantly of α cells 23, 31. Natural cell arrangement could also be detected in human islet organoids, but that was uncontrollable 15, 33. To mimic the natural topological arrangement, some hypotheses were proposed, such as abiding by sequential and timing incorporation of tissue- and organ-specific cell progenitors when organoid took shape, which could simulate the organ development to a greater extent 168. Moreover, 3D-bioprinting is supposed to be the best solution with premise of higher accuracy, since it can precisely print each cell to the designated location. The different kinds of cells intermingled with bioink which supplemented with corresponding specific growth factors during the islet organoids bioprinting, also be expected to recreate the natural topological cell arrangement of human islet.

**The high heterogeneity of human islet organoids.** Nowadays, many efforts have been made to solve the heterogeneity of islet organoids. The robust differentiation protocol to obtain relatively stable and consistent islet cells in different cell lines has been invented, and the heterogeneity of organoid size has been reduced by microwell platform and bioprinting 62. However, the phenotypic variation of islet organoids (e.g., viability and shape) and their responses to external stimulations are of concern. Many molecules or matrices applied in organoid generation with unknown mechanisms would give rise to the heterogeneity, and the complex spatiotemporal dynamics of islet organoids generation are difficult to describe or predict 169. Thus, the regulation mechanism of human islet development must be understood. And as aforementioned, effective biomaterials with precise composition are supposed to be a robust inducer of islet organoids. Based on this, mathematical and computational models are applied to investigate organoid's heterogeneity by studying cellular interconnections and dynamics. For example, mathematical and computational models can analyze the distribution of cell populations and growth patterns in organoids by mimicking the signaling pathways dynamics and the nutrients consumption 170. From a mathematical modeling perspective, an organoid is a complex biological system whose dynamics and physiology can be predicted. Up to now, mathematical modeling of optic-cup organoids, cerebral organoids and intestinal organoids have been created 171-173. Yet there is still no mathematical modeling of pancreatic or islet organoids, which needs to be elucidated in future studies, for the large scale of islet-like homogeneous organoids production.

**Long-term survival of human islet organoid**. Low sustainability is one of the biggest obstacles of human islet organoids development. During *in vitro* culture, lack of physical supports, oxygen and nutrients are main reasons for the instability of human islet organoids. Biomaterial is the most suitable solution for physical supports, and it can reduce cell apoptosis and functional impairment caused by hypoxia 174. Decreasing the size of islet organoids is another effective method to solve the instability caused by hypoxia and malnutrition. Moreover, a perfusive flow facilitating transport of nutrients, oxygen and waste introduced by microfluidic platform is also a feasible solution for islet organoids' *in vitro* survival.

After transplantation, immune rejection and low oxygen are main limitations for human islet organoids' long-term survival. In order to reduce the immune response, the solutions for immune tolerance are generally divided into two categories: graft modification and host regulation. Encapsulation with biomaterials is a promising strategy for avoiding immune rejection, which already has received lots of attentions 121, 122. Gene modifications in the transplanted islet organoids could also be the way to induce immune tolerance. With the sustained, endogenous expression of PD-L1 by CRISPR/Cas9, immune-evasive human islet-like organoids were generated by Yoshihara and colleagues recently 57. Employment of patients' self-derived iPSC or stringently selected HLA-homozygous iPSC lines as organoid cell resource has gradually led the new trend. In addition, transplant preconditioning is also the effective solution for immune protection. Host can be treated with immunosuppressive drugs, immune cell depletions as well as Treg-based adoptive cell therapies to induce persistent graft tolerance, as summarized by a couple of other reviews 69, 175.

*In vivo* hypoxia could cause apoptosis of islet cells, impairment of glucose response and insulin secretion. To increase the oxygen supply, there are a variety of solutions available: (1) generating pre-vascularization islet organoids by endothelial cells or potent angiogenic factors, and connecting them with host blood vessels after transplantation 176. This strategy has been described in detail by Nazeer and colleagues 177; (2) embedding islets into macro-porous hydrogel constructs made by 3D extrusion bioprinting to reduce the distance between islets and the surrounding body fluid/blood vessels 178; (3) adding oxygen-releasing device into islets transplantation platform to provide a stable source of oxygen, such as oxygen pump. For example, a macro-porous heparin-releasing silk fibroin scaffold has been proved to promote islet re-vascularization and proliferation by heparin-dependent activation of endogenous VEGF/VEGFR2 pathway 179. At present, a transplantation product called βAir for T1D treatment has been designed in which islets are encapsulated with alginate hydrogel, which serves as a scaffold for islet immobilization and protecting islets from the immune system. In this device, oxygen is supplied by the oxygen tank compartment which is fed with oxygen periodically 180.

Despite there are still some imperfections in islet organoid technology, islet organoid systems have already been proven to promote the studies of human islet, especially in the field of human islet development, diabetic disease model and islet transplantation 12, 181. Nearly two decades have passed since the first success of the Edmonton protocol, the cadaveric islets have not been the only resource for transplantation. Stem cell-based clinical trials for diabetes have been proposed and performed in recent years 182. The first trial for T1D treated by human ESC-derived pancreatic progenitor transplantation was submitted by ViaCyte in 2014 (NCT02239354). In January 2021, a clinical trial of hESC-derived β cell therapy for T1D has been approved for IND, launched by Semma Therapeutics/Vertex (NCT04786262). Recently, the interim results of a phase I/II clinical trial (NCT03163511) have been reported by ViaCyte, in which human pancreatic endoderm cells were implanted in macroencapsulation device for T1D treatment. This study recruited 26 T1D patients and detected the safety, tolerability and efficacy of this combination product (VC-02TM) up to 1 year after transplantation. The engraftment was detectable between 3 to 12 months after transplantation in the majority of patients with no teratoma formation or severe graft-related adverse events. The increased C-peptide and insulin secretion was observed and meal-induced C-peptide concentrations were found to increase at 26 and 52 weeks after implantation 183, 184. This is a promising encouragement for human islet organoid development. Thus, the booming human islet regeneration will undoubtedly bring regeneration medicine closer to diabetic patients and be expected to replenish the clinical transplantation needs of cadaveric islets.

## Figures and Tables

**Table 1:** Summary of representative achievements in making human islet organoids.

Published Year	Cell Resource	Materials	3D Structure Constitution	Reference
2001	hESC forming 3D structure, followed with spontaneous differentiation	-	-	16
2007	IPC derived from hESC	-	Self-clustering in hydrophobic culture dishes	17
2008	PP derived from hiPSC	-	Self-clustering in ultra-low-attachment plastic plates	18
2012	Human 1.1B4 β-cell line	-	Self-clustering in ultra-low-attachment plastic plates	19
2014	PP digested from human fetal pancreas, and liver stromal cells derived from human fetal liver	-	Self-clustering in non-adherent plates	20
2015	PP derived from hESC	PEG and Collagen I	Encapsulated in PEG-collagen I hydrogel	21
	Human 1.1B4 β-cell line	-	Self-clustering in ultra-low-attachment plate by centrifugation	22
	Dispersing human islet cells	-	Self-clustering in agarose microwell platform	23
	PE from human pancreas with lentiviruses expressing MAPK and STAT3	Matrigel	Self-clustering in suspension culture or embedded in Matrigel	24
	IPC derived from hiPSC	Gelatin	Self-clustering in gelatin-coated plates, then vascularizing by transplantation	25
	PE derived from hESC	-	PE clusters on 2D adherent culture were enriched by hand-picking, then cultured in suspension culture	26
2016	Human 1.1B4 β-cell line	-	Self-clustering in ultra-low-attachment plastic plates	27
	PE derived from hESC	-	Self-clustering in ultra-low-attachment plastic plates	28
	Dispersing human islet cells	Silk foam	Self-clustering in silk foam	29
2017	hPSC forming 3D structure, followed with pancreatic differentiation	-	Self-clustering in agarose microwell platform	30
	Dispersing human islet cells and human mesenchymal stromal cells	Alginate and PEG	Self-clustering by hanging-drop, then encapsulated in Alginate-PEG hydrogel for transplantation	31
	hPSC forming 3D structure, followed with pancreatic differentiation	Collagen I and Matrigel	Embedded with collagen I-Matrigel hydrogel	32
	Dispersing human islet cells	-	Self-clustering by hanging-drop	33
2018	hiPSC forming 3D structure, followed with pancreatic differentiation	PCL/PVA	Self-clustering in non-adherent plates, then seeded on PCL/PVA nanofiber scaffolds	34
	PP derived from hESC, HUVEC	Amikagel	Self-clustering on Amikagel	35
	hiPSC forming 3D structure, followed with pancreatic differentiation	PLLA/PVA	Self-clustering in non-adherent plates, then seeded on PLLA/PVA nanofiber scaffolds	36
	hESC transfected with synRNA-PDX1 and synRNA-NKX6.1	-	Self-clustering in ultra-low-attachment plastic plates	37
	Small pieces of exocrine tissue from human adult islet-depleted pancreatic tissue	Matrigel	Embedded in Matrigel	38
	Human EndoC-βH1 β-cell and endothelial cell	-	Self-clustering in ultra-low-attachment plastic plates	39
	Dispersing human islet cells	-	Self-clustering in AggreWell by centrifugation	40
2019	IPC derived from hESC, and HUVEC	Matrigel	Self-clustering on Matrigel-coated plate	41
	hiPSC forming 3D structure, followed with islet differentiation	Alginate	Self-clustering in spinning flasks, then encapsulated with alginate fiber for transplantation	42
	IPC derived from hPSCs, and HUVEC	Alginate, Collagen or Porcine pancreatic dECM as bioink	Embedded in bioink, then followed with 3D cell printing	43
	Dispersing human islet cells and human amniotic epithelial cells	-	Self-clustering in agarose microwell platform	44
	Human liver stem-like Cells forming 3D structure, followed with islet differentiation	-	Self-clustering in flasks with protamine chloride	45
	hESC forming 3D structure, followed with islet differentiation	-	Self-clustering in spinning flasks	15
	PP, MSC, and endothelial cells derived from hESC	Matrigel or Agarose and PLA	Self-clustering in Matrigel- or agarose-coated plate, then put in PLA basket‐like scaffold by 3D printing	46
	hiPSC forming 3D structure, followed with islet differentiation	-	Self-clustering in microwell by centrifugation, then cultured in multi-layer microfluidic microsystem	47
	hiPSCs forming 3D structure, followed with islet differentiation	-	Self-clustering in spinning flasks	48
	DE from hiPSCs followed with islet differentiation	-	Self-clustering on chip with optimal topographical structure.	49.
2020	PP derived from hiPSCs followed with islet differentiation	Matrigel and rat pancreatic dECM	Self-clustering in ultra-low-attachment plastic plates, then coated with Matrigel and rat pancreatic dECM	50
	PE derived from hiPSC on Matrigel, and human collagen V coated plates	Matrigel and human collagen V	Self-clustering in ultra-low-attachment plastic plates	51
	PE derived from hiPSC	Alginate	Embedded in alginate	52
	PE derived from hiPSC	Chitosan-coated Alginate	Encapsulated in chitosan-coated alginate by droplet microfluidic system	53
	IPC derived from hPSC	PEG-MAL and SH-PEG-SH	Self-clustering in spinning flasks, then followed with PEG-MAL and SH-PEG-SH encapsulated	54
	Human EndoC-βH1 β-cells and HUVEC	-	Self-clustering in low adherence U-bottom plates	55
	Dispersing human islet cells with efficient virally mediated genetic manipulation	-	Self-clustering by hanging drop and ultra-low attachment microwell, then cultured in a microfluidic system	56
	PEP derived from hiPSC with PD-L1 overexpression, HUVEC and hADSC	Matrigel	Self-clustering on Matrigel coated plate	57
2021	PEP derived from hiPSC	Sodium alginate, Calcium chloride and PEG	Encapsulated within microfibers, made by sodium alginate, calcium chloride and PEG	58
	PE derived from hiPSC, followed with islet differentiation and angiopoietins for endothelium formation	-	Self-clustering in ultra-low-attachment plastic plates	59
	hESC forming 3D structure, followed with islet differentiation	Laminin, nidogen and collagen IV	Self-clustering in stirring bioreactor then coating with proteins	60
	Dispersing human islet cells	-	Self-clustering by hanging-drop,	61
2021	PE derived from hiPSC, followed with islet differentiation	-	Self-clustering in plate on orbital shaker	62
	PE derived from hiPSC, followed with islet differentiation	-	Self-clustering in plate on orbital shaker and cultured in a dialysis suspension culture system	63
	IPC derived from hESC,	Porcine pancreatic dECM and PCL	Embedded in dECM bioink, then 3D bioprinted into PCL scaffold	64

hESC: human embryonic stem cell; hiPSC: human induced pluripotent stem cell; hPSC: human pluripotent stem cell; PP: pancreatic progenitor; PE: pancreatic endocrine cell; IPC: insulin-producing cell; HUVEC: human umbilical vein endothelial cell; MSC: mesenchymal stem cell; dECM: decellularization extracellular matrix; PEP: pancreatic endocrine progenitor; hADSC: human adipose-derived stem cell; DE: definitive endodermal cell.

**Table 2:** Comparison of natural and synthetic materials supplied for human islet organoids generation.

	Natural matrix biomaterial	Synthetic matrix material
**Classification**	Polysaccharide-based: alginate, chitosan, agarose, etc.	PEG, PLA, PLGA, PVA, PCL, etc.
	Protein/peptide-based: collagen, gelatin, etc.	
	dECM-based	
**Advantages**	Cell adhesion and cell interaction ability; Tissue-specific composition (specific growth factors, mechanical properties)	Reproducibility and stability; Precise and tunable mechanical conditions; Highly economic value
**Limitation**	May introduce cell-dedifferentiation;	Low cell adhesion and cell interaction ability
	Time-consuming and costly;	
	Batch-to-batch variability;	
	Poor mechanical properties and insufficient stability	

## References

[1] Larsen HL, Grapin-Botton A (2017). The molecular and morphogenetic basis of pancreas organogenesis. Semin Cell Dev Biol.

[2] Zhang J, Liu F (2020). The De-, Re-, and trans-differentiation of beta-cells: Regulation and function. Semin Cell Dev Biol.

[3] Yuen L, Saeedi P, Riaz M, Karuranga S, Divakar H, Levitt N (2019). Projections of the prevalence of hyperglycaemia in pregnancy in 2019 and beyond: Results from the International Diabetes Federation Diabetes Atlas, 9th edition. Diabetes Res Clin Pract.

[4] Atkinson MA, Eisenbarth GS, Michels AW (2014). Type 1 diabetes. Lancet.

[5] Verhoeff K, Henschke SJ, Marfil-Garza BA, Dadheech N, Shapiro AMJ (2021). Inducible Pluripotent Stem Cells as a Potential Cure for Diabetes. Cells.

[6] Shapiro AM, Lakey JR, Ryan EA, Korbutt GS, Toth E, Warnock GL (2000). Islet transplantation in seven patients with type 1 diabetes mellitus using a glucocorticoid-free immunosuppressive regimen. N Engl J Med.

[7] Markmann JF, Rickels MR, Eggerman TL, Bridges ND, Lafontant DE, Qidwai J (2020). Phase 3 Trial of Human Islet-after-Kidney Transplantation in Type 1 Diabetes. Am J Transplant.

[8] Lancaster MA, Knoblich JA (2014). Organogenesis in a dish: modeling development and disease using organoid technologies. Science.

[9] Sato T, Vries RG, Snippert HJ, van de Wetering M, Barker N, Stange DE (2009). Single Lgr5 stem cells build crypt-villus structures in vitro without a mesenchymal niche. Nature.

[10] Clevers H (2016). Modeling Development and Disease with Organoids. Cell.

[11] Schutgens F, Clevers H (2020). Human Organoids: Tools for Understanding Biology and Treating Diseases. Annu Rev Pathol.

[12] Zhang X, Ma Z, Song E, Xu T (2021). Islet organoid as a promising model for diabetes. Protein Cell.

[13] Wassmer CH, Lebreton F, Bellofatto K, Bosco D, Berney T, Berishvili E (2020). Generation of insulin-secreting organoids: a step toward engineering and transplanting the bioartificial pancreas. Transpl Int.

[14] Pan FC, Brissova M (2014). Pancreas development in humans. Curr Opin Endocrinol Diabetes Obes.

[15] Sharon N, Chawla R, Mueller J, Vanderhooft J, Whitehorn LJ, Rosenthal B (2019). A Peninsular Structure Coordinates Asynchronous Differentiation with Morphogenesis to Generate Pancreatic Islets. Cell.

[16] Assady S, Maor G, Amit M, Itskovitz-Eldor J, Skorecki KL, Tzukerman M (2001). Insulin production by human embryonic stem cells. Diabetes.

[17] Jiang W, Shi Y, Zhao D, Chen S, Yong J, Zhang J (2007). In vitro derivation of functional insulin-producing cells from human embryonic stem cells. Cell research.

[18] Tateishi K, He J, Taranova O, Liang G, D'Alessio AC, Zhang Y (2008). Generation of insulin-secreting islet-like clusters from human skin fibroblasts. J Biol Chem.

[19] Guo-Parke H, McCluskey JT, Kelly C, Hamid M, McClenaghan NH, Flatt PR (2012). Configuration of electrofusion-derived human insulin-secreting cell line as pseudoislets enhances functionality and therapeutic utility. J Endocrinol.

[20] Liang J, Ng KY, Cheng Q, Xia Y, Wang CC, Leung PS (2014). Human fetal liver stromal cell co-culture enhances the differentiation of pancreatic progenitor cells into islet-like cell clusters. Stem Cell Rev Rep.

[21] Amer LD, Holtzinger A, Keller G, Mahoney MJ, Bryant SJ (2015). Enzymatically degradable poly(ethylene glycol) hydrogels for the 3D culture and release of human embryonic stem cell derived pancreatic precursor cell aggregates. Acta Biomater.

[22] Green AD, Vasu S, McClenaghan NH, Flatt PR (2015). Pseudoislet formation enhances gene expression, insulin secretion and cytoprotective mechanisms of clonal human insulin-secreting 1.1B4 cells. Pflugers Arch.

[23] Hilderink J, Spijker S, Carlotti F, Lange L, Engelse M, van Blitterswijk C (2015). Controlled aggregation of primary human pancreatic islet cells leads to glucose-responsive pseudoislets comparable to native islets. J Cell Mol Med.

[24] Lemper M, Leuckx G, Heremans Y, German MS, Heimberg H, Bouwens L (2015). Reprogramming of human pancreatic exocrine cells to beta-like cells. Cell Death Differ.

[25] Raikwar SP, Kim EM, Sivitz WI, Allamargot C, Thedens DR, Zavazava N (2015). Human iPS cell-derived insulin producing cells form vascularized organoids under the kidney capsules of diabetic mice. PLoS One.

[26] Shim JH, Kim J, Han J, An SY, Jang YJ, Son J (2015). Pancreatic Islet-Like Three-Dimensional Aggregates Derived From Human Embryonic Stem Cells Ameliorate Hyperglycemia in Streptozotocin-Induced Diabetic Mice. Cell Transplant.

[27] Green AD, Vasu S, McClenaghan NH, Flatt PR (2016). Implanting 1.1B4 human beta-cell pseudoislets improves glycaemic control in diabetic severe combined immune deficient mice. World J Diabetes.

[28] Kim Y, Kim H, Ko UH, Oh Y, Lim A, Sohn JW (2016). Islet-like organoids derived from human pluripotent stem cells efficiently function in the glucose responsiveness in vitro and in vivo. Sci Rep.

[29] Shalaly ND, Ria M, Johansson U, Åvall K, Berggren PO, Hedhammar M (2016). Silk matrices promote formation of insulin-secreting islet-like clusters. Biomaterials.

[30] Hirano K, Konagaya S, Turner A, Noda Y, Kitamura S, Kotera H (2017). Closed-channel culture system for efficient and reproducible differentiation of human pluripotent stem cells into islet cells. Biochem Biophys Res Commun.

[31] Montanari E, Meier RPH, Mahou R, Seebach JD, Wandrey C, Gerber-Lemaire S (2017). Multipotent mesenchymal stromal cells enhance insulin secretion from human islets via N-cadherin interaction and prolong function of transplanted encapsulated islets in mice. Stem Cell Res Ther.

[32] Wang W, Jin S, Ye K (2017). Development of Islet Organoids from H9 Human Embryonic Stem Cells in Biomimetic 3D Scaffolds. Stem Cells Dev.

[33] Zuellig RA, Cavallari G, Gerber P, Tschopp O, Spinas GA, Moritz W (2017). Improved physiological properties of gravity-enforced reassembled rat and human pancreatic pseudo-islets. J Tissue Eng Regen Med.

[34] Abazari MF, Soleimanifar F, Nouri Aleagha M, Torabinejad S, Nasiri N, Khamisipour G (2018). PCL/PVA nanofibrous scaffold improve insulin-producing cells generation from human induced pluripotent stem cells. Gene.

[35] Candiello J, Grandhi TSP, Goh SK, Vaidya V, Lemmon-Kishi M, Eliato KR (2018). 3D heterogeneous islet organoid generation from human embryonic stem cells using a novel engineered hydrogel platform. Biomaterials.

[36] Enderami SE, Kehtari M, Abazari MF, Ghoraeian P, Nouri Aleagha M, Soleimanifar F (2018). Generation of insulin-producing cells from human induced pluripotent stem cells on PLLA/PVA nanofiber scaffold. Artif Cells Nanomed Biotechnol.

[37] Ida H, Akiyama T, Ishiguro K, Goparaju SK, Nakatake Y, Chikazawa-Nohtomi N (2018). Establishment of a rapid and footprint-free protocol for differentiation of human embryonic stem cells into pancreatic endocrine cells with synthetic mRNAs encoding transcription factors. Stem Cell Res Ther.

[38] Loomans CJM, Williams Giuliani N, Balak J, Ringnalda F, van Gurp L, Huch M (2018). Expansion of Adult Human Pancreatic Tissue Yields Organoids Harboring Progenitor Cells with Endocrine Differentiation Potential. Stem Cell Reports.

[39] Spelios MG, Afinowicz LA, Tipon RC, Akirav EM (2018). Human EndoC-βH1 β-cells form pseudoislets with improved glucose sensitivity and enhanced GLP-1 signaling in the presence of islet-derived endothelial cells. Am J Physiol Endocrinol Metab.

[40] Yu Y, Gamble A, Pawlick R, Pepper AR, Salama B, Toms D (2018). Bioengineered human pseudoislets form efficiently from donated tissue, compare favourably with native islets in vitro and restore normoglycaemia in mice. Diabetologia.

[41] Augsornworawat P, Velazco-Cruz L, Song J, Millman JR (2019). A hydrogel platform for in vitro three dimensional assembly of human stem cell-derived islet cells and endothelial cells. Acta Biomater.

[42] Fukuda S, Yabe SG, Nishida J, Takeda F, Nashiro K, Okochi H (2019). The intraperitoneal space is more favorable than the subcutaneous one for transplanting alginate fiber containing iPS-derived islet-like cells. Regen Ther.

[43] Kim J, Shim IK, Hwang DG, Lee YN, Kim M, Kim H (2019). 3D cell printing of islet-laden pancreatic tissue-derived extracellular matrix bioink constructs for enhancing pancreatic functions. J Mater Chem B.

[44] Lebreton F, Lavallard V, Bellofatto K, Bonnet R, Wassmer CH, Perez L (2019). Insulin-producing organoids engineered from islet and amniotic epithelial cells to treat diabetes. Nat Commun.

[45] Navarro-Tableros V, Gai C, Gomez Y, Giunti S, Pasquino C, Deregibus MC (2019). Islet-Like Structures Generated In Vitro from Adult Human Liver Stem Cells Revert Hyperglycemia in Diabetic SCID Mice. Stem Cell Rev Rep.

[46] Soltanian A, Ghezelayagh Z, Mazidi Z, Halvaei M, Mardpour S, Ashtiani MK (2019). Generation of functional human pancreatic organoids by transplants of embryonic stem cell derivatives in a 3D-printed tissue trapper. J Cell Physiol.

[47] Tao T, Wang Y, Chen W, Li Z, Su W, Guo Y (2019). Engineering human islet organoids from iPSCs using an organ-on-chip platform. Lab Chip.

[48] Yabe SG, Fukuda S, Nishida J, Takeda F, Nashiro K, Okochi H (2019). Induction of functional islet-like cells from human iPS cells by suspension culture. Regen Ther.

[49] Kim JH, Park BG, Kim SK, Lee DH, Lee GG, Kim DH (2019). Nanotopographical regulation of pancreatic islet-like cluster formation from human pluripotent stem cells using a gradient-pattern chip. Acta Biomater.

[50] Bi H, Karanth SS, Ye K, Stein R, Jin S (2020). Decellularized Tissue Matrix Enhances Self-Assembly of Islet Organoids from Pluripotent Stem Cell Differentiation. ACS biomaterials science & engineering.

[51] Bi H, Ye K, Jin S (2020). Proteomic analysis of decellularized pancreatic matrix identifies collagen V as a critical regulator for islet organogenesis from human pluripotent stem cells. Biomaterials.

[52] Legoy TA, Vethe H, Abadpour S, Strand BL, Scholz H, Paulo JA (2020). Encapsulation boosts islet-cell signature in differentiating human induced pluripotent stem cells via integrin signalling. Sci Rep.

[53] Liu H, Wang Y, Wang H, Zhao M, Tao T, Zhang X (2020). A Droplet Microfluidic System to Fabricate Hybrid Capsules Enabling Stem Cell Organoid Engineering. Adv Sci (Weinh).

[54] Stock AA, Manzoli V, De Toni T, Abreu MM, Poh YC, Ye L (2020). Conformal Coating of Stem Cell-Derived Islets for beta Cell Replacement in Type 1 Diabetes. Stem Cell Reports.

[55] Urbanczyk M, Zbinden A, Layland SL, Duffy G, Schenke-Layland K (2020). Controlled Heterotypic Pseudo-Islet Assembly of Human β-Cells and Human Umbilical Vein Endothelial Cells Using Magnetic Levitation. Tissue Eng Part A.

[56] Walker JT, Haliyur R, Nelson HA, Ishahak M, Poffenberger G, Aramandla R (2020). Integrated human pseudoislet system and microfluidic platform demonstrate differences in GPCR signaling in islet cells. JCI Insight.

[57] Yoshihara E, O'Connor C, Gasser E, Wei Z, Oh TG, Tseng TW (2020). Immune-evasive human islet-like organoids ameliorate diabetes. Nature.

[58] Wang H, Liu H, Zhang X, Wang Y, Zhao M, Chen W (2021). One-Step Generation of Aqueous-Droplet-Filled Hydrogel Fibers as Organoid Carriers Using an All-in-Water Microfluidic System. ACS Appl Mater Interfaces.

[59] Karanth SS, Sun S, Bi H, Ye K, Jin S (2021). Angiopoietins stimulate pancreatic islet development from stem cells. Sci Rep.

[60] Santini-Gonzalez J, Simonovich JA, Castro-Gutierrez R, Gonzalez-Vargas Y, Abuid NJ, Stabler CL (2021). In vitro generation of peri-islet basement membrane-like structures. Biomaterials.

[61] Mir-Coll J, Moede T, Paschen M, Neelakandhan A, Valladolid-Acebes I, Leibiger B (2021). Human Islet Microtissues as an In Vitro and an In Vivo Model System for Diabetes. Int J Mol Sci.

[62] Hogrebe NJ, Maxwell KG, Augsornworawat P, Millman JR (2021). Generation of insulin-producing pancreatic beta cells from multiple human stem cell lines. Nat Protoc.

[63] Choi H, Shinohara M, Ibuki M, Nishikawa M, Sakai Y (2021). Differentiation of Human-Induced Pluripotent Stem Cell-Derived Endocrine Progenitors to Islet-like Cells Using a Dialysis Suspension Culture System. Cells.

[64] Hwang DG, Jo Y, Kim M, Yong U, Cho S, Choi YM (2021). A 3D bioprinted hybrid encapsulation system for delivery of human pluripotent stem cell-derived pancreatic islet-like aggregates. Biofabrication.

[65] Yamanaka S (2020). Pluripotent Stem Cell-Based Cell Therapy-Promise and Challenges. Cell stem cell.

[66] Gaertner B, Carrano AC, Sander M (2019). Human stem cell models: lessons for pancreatic development and disease. Genes Dev.

[67] Balboa D, Iworima DG, Kieffer TJ (2021). Human Pluripotent Stem Cells to Model Islet Defects in Diabetes. Front Endocrinol (Lausanne).

[68] Helman A, Melton DA (2021). A Stem Cell Approach to Cure Type 1 Diabetes. Cold Spring Harb Perspect Biol.

[69] Tahbaz M, Yoshihara E (2021). Immune Protection of Stem Cell-Derived Islet Cell Therapy for Treating Diabetes. Front Endocrinol (Lausanne).

[70] Migliorini A, Nostro MC, Sneddon JB (2021). Human pluripotent stem cell-derived insulin-producing cells: A regenerative medicine perspective. Cell Metab.

[71] Maxwell KG, Millman JR (2021). Applications of iPSC-derived beta cells from patients with diabetes. Cell Rep Med.

[72] Zhang D, Jiang W, Liu M, Sui X, Yin X, Chen S (2009). Highly efficient differentiation of human ES cells and iPS cells into mature pancreatic insulin-producing cells. Cell Res.

[73] Sahabian A, Sgodda M, Naujok O, Dettmer R, Dahlmann J, Manstein F (2019). Chemically-Defined, Xeno-Free, Scalable Production of hPSC-Derived Definitive Endoderm Aggregates with Multi-Lineage Differentiation Potential. Cells.

[74] Konagaya S, Iwata H (2019). Chemically defined conditions for long-term maintenance of pancreatic progenitors derived from human induced pluripotent stem cells. Sci Rep.

[75] Wang Z, York NW, Nichols CG, Remedi MS (2014). Pancreatic β cell dedifferentiation in diabetes and redifferentiation following insulin therapy. Cell Metab.

[76] Aloy-Reverté C, Moreno-Amador JL, Nacher M, Montanya E, Semino CE (2018). Use of RGD-Functionalized Sandwich Cultures to Promote Redifferentiation of Human Pancreatic Beta Cells After In Vitro Expansion. Tissue Eng Part A.

[77] Ariyachet C, Tovaglieri A, Xiang G, Lu J, Shah MS, Richmond CA (2016). Reprogrammed Stomach Tissue as a Renewable Source of Functional β Cells for Blood Glucose Regulation. Cell Stem Cell.

[78] Wang D, Wang J, Bai L, Pan H, Feng H, Clevers H (2020). Long-Term Expansion of Pancreatic Islet Organoids from Resident Procr(+) Progenitors. Cell.

[79] Narayanan S, Loganathan G, Dhanasekaran M, Tucker W, Patel A, Subhashree V (2017). Intra-islet endothelial cell and beta-cell crosstalk: Implication for islet cell transplantation. World J Transplant.

[80] Guney MA, Petersen CP, Boustani A, Duncan MR, Gunasekaran U, Menon R (2011). Connective tissue growth factor acts within both endothelial cells and beta cells to promote proliferation of developing beta cells. Proceedings of the National Academy of Sciences of the United States of America.

[81] Talavera-Adame D, Woolcott OO, Ignatius-Irudayam J, Arumugaswami V, Geller DH, Dafoe DC (2016). Effective endothelial cell and human pluripotent stem cell interactions generate functional insulin-producing beta cells. Diabetologia.

[82] Jaramillo M, Singh SS, Velankar S, Kumta PN, Banerjee I (2015). Inducing endoderm differentiation by modulating mechanical properties of soft substrates. J Tissue Eng Regen Med.

[83] Takebe T, Sekine K, Enomura M, Koike H, Kimura M, Ogaeri T (2013). Vascularized and functional human liver from an iPSC-derived organ bud transplant. Nature.

[84] Takebe T, Enomura M, Yoshizawa E, Kimura M, Koike H, Ueno Y (2015). Vascularized and Complex Organ Buds from Diverse Tissues via Mesenchymal Cell-Driven Condensation. Cell Stem Cell.

[85] Takahashi Y, Sekine K, Kin T, Takebe T, Taniguchi H (2018). Self-Condensation Culture Enables Vascularization of Tissue Fragments for Efficient Therapeutic Transplantation. Cell Rep.

[86] Fransson M, Brannstrom J, Duprez I, Essand M, Le Blanc K, Korsgren O (2015). Mesenchymal stromal cells support endothelial cell interactions in an intramuscular islet transplantation model. Regen Med Res.

[87] Bal T, Nazli C, Okcu A, Duruksu G, Karaöz E, Kizilel S (2017). Mesenchymal stem cells and ligand incorporation in biomimetic poly(ethylene glycol) hydrogels significantly improve insulin secretion from pancreatic islets. J Tissue Eng Regen Med.

[88] Akolpoglu MB, Inceoglu Y, Bozuyuk U, Sousa AR, Oliveira MB, Mano JF (2021). Recent advances in the design of implantable insulin secreting heterocellular islet organoids. Biomaterials.

[89] Liu H, Wang Y, Cui K, Guo Y, Zhang X, Qin J (2019). Advances in Hydrogels in Organoids and Organs-on-a-Chip. Adv Mater.

[90] Liaw CY, Ji S, Guvendiren M (2018). Engineering 3D Hydrogels for Personalized In Vitro Human Tissue Models. Adv Healthc Mater.

[91] Lim F, Sun AM (1980). Microencapsulated islets as bioartificial endocrine pancreas. Science.

[92] Xu L, Guo Y, Huang Y, Xu Y, Lu Y, Wang Z (2019). Hydrogel materials for the application of islet transplantation. J Biomater Appl.

[93] Bao W, Li M, Yang Y, Wan Y, Wang X, Bi N (2020). Advancements and Frontiers in the High Performance of Natural Hydrogels for Cartilage Tissue Engineering. Front Chem.

[94] Canibano-Hernandez A, Saenz Del Burgo L, Espona-Noguera A, Orive G, Hernandez RM, Ciriza J (2019). Hyaluronic acid enhances cell survival of encapsulated insulin-producing cells in alginate-based microcapsules. Int J Pharm.

[95] Cañibano-Hernández A, Saenz Del Burgo L, Espona-Noguera A, Orive G, Hernández RM, Ciriza J (2019). Hyaluronic Acid Promotes Differentiation of Mesenchymal Stem Cells from Different Sources toward Pancreatic Progenitors within Three-Dimensional Alginate Matrixes. Mol Pharm.

[96] Su X, Tan M, Duan B, Cai J, Jiang W, Zhang L (2019). Hierarchical microspheres with macropores fabricated from chitin as 3D cell culture. J Mater Chem B.

[97] Wang X, Ye K (2009). Three-dimensional differentiation of embryonic stem cells into islet-like insulin-producing clusters. Tissue Eng Part A.

[98] Riopel M, Wang R (2014). Collagen matrix support of pancreatic islet survival and function. Front Biosci (Landmark Ed).

[99] Daoud J, Petropavlovskaia M, Rosenberg L, Tabrizian M (2010). The effect of extracellular matrix components on the preservation of human islet function in vitro. Biomaterials.

[100] Kim BS, Kim H, Gao G, Jang J, Cho DW (2017). Decellularized extracellular matrix: a step towards the next generation source for bioink manufacturing. Biofabrication.

[101] Giobbe GG, Crowley C, Luni C, Campinoti S, Khedr M, Kretzschmar K (2019). Extracellular matrix hydrogel derived from decellularized tissues enables endodermal organoid culture. Nat Commun.

[102] De Carlo E, Baiguera S, Conconi MT, Vigolo S, Grandi C, Lora S (2010). Pancreatic acellular matrix supports islet survival and function in a synthetic tubular device: in vitro and in vivo studies. Int J Mol Med.

[103] Wu D, Wan J, Huang Y, Guo Y, Xu T, Zhu M (2015). 3D Culture of MIN-6 Cells on Decellularized Pancreatic Scaffold: In Vitro and In Vivo Study. Biomed Res Int.

[104] Narayanan K, Lim VY, Shen J, Tan ZW, Rajendran D, Luo SC (2014). Extracellular matrix-mediated differentiation of human embryonic stem cells: differentiation to insulin-secreting beta cells. Tissue Eng Part A.

[105] Czerwinski M, Spence JR (2017). Hacking the Matrix. Cell stem cell.

[106] Zhang M, Yan S, Xu X, Yu T, Guo Z, Ma M (2021). Three-dimensional cell-culture platform based on hydrogel with tunable microenvironmental properties to improve insulin-secreting function of MIN6 cells. Biomaterials.

[107] Mao GH, Chen GA, Bai HY, Song TR, Wang YX (2009). The reversal of hyperglycaemia in diabetic mice using PLGA scaffolds seeded with islet-like cells derived from human embryonic stem cells. Biomaterials.

[108] Kuo YC, Liu YC, Rajesh R (2017). Pancreatic differentiation of induced pluripotent stem cells in activin A-grafted gelatin-poly(lactide-co-glycolide) nanoparticle scaffolds with induction of LY294002 and retinoic acid. Mater Sci Eng C Mater Biol Appl.

[109] Song J, Millman JR (2016). Economic 3D-printing approach for transplantation of human stem cell-derived beta-like cells. Biofabrication.

[110] Zhang Y, Jalili RB, Warnock GL, Ao Z, Marzban L, Ghahary A (2012). Three-dimensional scaffolds reduce islet amyloid formation and enhance survival and function of cultured human islets. Am J Pathol.

[111] Takeichi M (2011). Self-organization of animal tissues: cadherin-mediated processes. Dev Cell.

[112] Choi YY, Chung BG, Lee DH, Khademhosseini A, Kim JH, Lee SH (2010). Controlled-size embryoid body formation in concave microwell arrays. Biomaterials.

[113] Lehmann R, Zuellig RA, Kugelmeier P, Baenninger PB, Moritz W, Perren A (2007). Superiority of small islets in human islet transplantation. Diabetes.

[114] Mendelsohn AD, Bernards DA, Lowe RD, Desai TA (2010). Patterning of mono- and multilayered pancreatic beta-cell clusters. Langmuir.

[115] Brassard JA, Lutolf MP (2019). Engineering Stem Cell Self-organization to Build Better Organoids. Cell Stem Cell.

[116] Ichihara Y, Utoh R, Yamada M, Shimizu T, Uchigata Y (2016). Size effect of engineered islets prepared using microfabricated wells on islet cell function and arrangement. Heliyon.

[117] Lee G, Jun Y, Jang H, Yoon J, Lee J, Hong M (2018). Enhanced oxygen permeability in membrane-bottomed concave microwells for the formation of pancreatic islet spheroids. Acta Biomater.

[118] Hadavi E, Leijten J, Engelse M, de Koning E, Jonkheijm P, Karperien M (2019). Microwell Scaffolds Using Collagen-IV and Laminin-111 Lead to Improved Insulin Secretion of Human Islets. Tissue Eng Part C Methods.

[119] Gao B, Wang L, Han S, Pingguan-Murphy B, Zhang X, Xu F (2016). Engineering of microscale three-dimensional pancreatic islet models in vitro and their biomedical applications. Crit Rev Biotechnol.

[120] Korsgren O (2017). Islet Encapsulation: Physiological Possibilities and Limitations. Diabetes.

[121] Marchioli G, Luca AD, de Koning E, Engelse M, Van Blitterswijk CA, Karperien M (2016). Hybrid Polycaprolactone/Alginate Scaffolds Functionalized with VEGF to Promote de Novo Vessel Formation for the Transplantation of Islets of Langerhans. Adv Healthc Mater.

[122] Bukys MA, Bakos B, Afelik S, Zimmerman B, Barbaro B, Lin DL (2016). Xeno-Transplantation of macro-encapsulated islets and Pluripotent Stem Cell-Derived Pancreatic Progenitors without Immunosuppression. J Stem Cell Transplant Biol.

[123] Jiang K, Chaimov D, Patel SN, Liang JP, Wiggins SC, Samojlik MM (2019). 3-D physiomimetic extracellular matrix hydrogels provide a supportive microenvironment for rodent and human islet culture. Biomaterials.

[124] Johansson U, Ria M, Åvall K, Dekki Shalaly N, Zaitsev SV, Berggren PO (2015). Pancreatic Islet Survival and Engraftment Is Promoted by Culture on Functionalized Spider Silk Matrices. PLoS One.

[125] Derby B (2012). Printing and prototyping of tissues and scaffolds. Science.

[126] Kim J, Kang K, Drogemuller CJ, Wallace GG, Coates PT (2019). Bioprinting an Artificial Pancreas for Type 1 Diabetes. Current diabetes reports.

[127] Yue Z, Liu X, Coates PT, Wallace GG (2016). Advances in printing biomaterials and living cells: implications for islet cell transplantation. Curr Opin Organ Transplant.

[128] Duin S, Schütz K, Ahlfeld T, Lehmann S, Lode A, Ludwig B (2019). 3D Bioprinting of Functional Islets of Langerhans in an Alginate/Methylcellulose Hydrogel Blend. Adv Healthc Mater.

[129] Espona-Noguera A, Ciriza J, Canibano-Hernandez A, Orive G, Hernandez RMM, Saenz Del Burgo L (2019). Review of Advanced Hydrogel-Based Cell Encapsulation Systems for Insulin Delivery in Type 1 Diabetes Mellitus. Pharmaceutics.

[130] Liu X, Carter SD, Renes MJ, Kim J, Rojas-Canales DM, Penko D (2019). Development of a Coaxial 3D Printing Platform for Biofabrication of Implantable Islet-Containing Constructs. Adv Healthc Mater.

[131] Bishop ES, Mostafa S, Pakvasa M, Luu HH, Lee MJ, Wolf JM (2017). 3-D bioprinting technologies in tissue engineering and regenerative medicine: Current and future trends. Genes Dis.

[132] Goh SK, Bertera S, Olsen P, Candiello JE, Halfter W, Uechi G (2013). Perfusion-decellularized pancreas as a natural 3D scaffold for pancreatic tissue and whole organ engineering. Biomaterials.

[133] Mirmalek-Sani SH, Orlando G, McQuilling JP, Pareta R, Mack DL, Salvatori M (2013). Porcine pancreas extracellular matrix as a platform for endocrine pancreas bioengineering. Biomaterials.

[134] Napierala H, Hillebrandt KH, Haep N, Tang P, Tintemann M, Gassner J (2017). Engineering an endocrine Neo-Pancreas by repopulation of a decellularized rat pancreas with islets of Langerhans. Sci Rep.

[135] Everwien H, Keshi E, Hillebrandt KH, Ludwig B, Weinhart M, Tang P (2020). Engineering an endothelialized, endocrine Neo-Pancreas: Evaluation of islet functionality in an ex vivo model. Acta Biomater.

[136] Wang Y, Wang H, Deng P, Chen W, Guo Y, Tao T (2018). In situ differentiation and generation of functional liver organoids from human iPSCs in a 3D perfusable chip system. Lab Chip.

[137] Takebe T, Zhang B, Radisic M (2017). Synergistic Engineering: Organoids Meet Organs-on-a-Chip. Cell Stem Cell.

[138] Patel SN, Ishahak M, Chaimov D, Velraj A, LaShoto D, Hagan DW (2021). Organoid microphysiological system preserves pancreatic islet function within 3D matrix. Sci Adv.

[139] Oleaga C, Bernabini C, Smith AS, Srinivasan B, Jackson M, McLamb W (2016). Multi-Organ toxicity demonstration in a functional human in vitro system composed of four organs. Sci Rep.

[140] Bauer S, Wennberg Huldt C, Kanebratt KP, Durieux I, Gunne D, Andersson S (2017). Functional coupling of human pancreatic islets and liver spheroids on-a-chip: Towards a novel human ex vivo type 2 diabetes model. Sci Rep.

[141] Rogal J, Zbinden A, Schenke-Layland K, Loskill P (2019). Stem-cell based organ-on-a-chip models for diabetes research. Adv Drug Deliv Rev.

[142] Tsakmaki A, Fonseca Pedro P, Bewick GA (2020). Diabetes through a 3D lens: organoid models. Diabetologia.

[143] Amin J, Ramachandran K, Williams SJ, Lee A, Novikova L, Stehno-Bittel L (2016). A simple, reliable method for high-throughput screening for diabetes drugs using 3D β-cell spheroids. J Pharmacol Toxicol Methods.

[144] Jun Y, Lee J, Choi S, Yang JH, Sander M, Chung S (2019). In vivo-mimicking microfluidic perfusion culture of pancreatic islet spheroids. Sci Adv.

[145] Castiello FR, Heileman K, Tabrizian M (2016). Microfluidic perfusion systems for secretion fingerprint analysis of pancreatic islets: applications, challenges and opportunities. Lab Chip.

[146] Mamidi A, Prawiro C, Seymour PA, de Lichtenberg KH, Jackson A, Serup P (2018). Mechanosignalling via integrins directs fate decisions of pancreatic progenitors. Nature.

[147] Hogrebe NJ, Augsornworawat P, Maxwell KG, Velazco-Cruz L, Millman JR (2020). Targeting the cytoskeleton to direct pancreatic differentiation of human pluripotent stem cells. Nat Biotechnol.

[148] Galli A, Algerta M, Marciani P, Schulte C, Lenardi C, Milani P (2020). Shaping Pancreatic β-Cell Differentiation and Functioning: The Influence of Mechanotransduction. Cells.

[149] Galli A, Maffioli E, Sogne E, Moretti S, Di Cairano ES, Negri A (2018). Cluster-assembled zirconia substrates promote long-term differentiation and functioning of human islets of Langerhans. Sci Rep.

[150] Klak M, Kowalska P, Dobrzański T, Tymicki G, Cywoniuk P, Gomółka M (2021). Bionic Organs: Shear Forces Reduce Pancreatic Islet and Mammalian Cell Viability during the Process of 3D Bioprinting. Micromachines (Basel).

[151] Pagliuca FW, Millman JR, Gurtler M, Segel M, Van Dervort A, Ryu JH (2014). Generation of functional human pancreatic beta cells in vitro. Cell.

[152] Rezania A, Bruin JE, Arora P, Rubin A, Batushansky I, Asadi A (2014). Reversal of diabetes with insulin-producing cells derived in vitro from human pluripotent stem cells. Nat Biotechnol.

[153] Huang H, Bader TN, Jin S (2020). Signaling Molecules Regulating Pancreatic Endocrine Development from Pluripotent Stem Cell Differentiation. Int J Mol Sci.

[154] Tan M, Jiang L, Li Y, Jiang W (2019). Dual Inhibition of BMP and WNT Signals Promotes Pancreatic Differentiation from Human Pluripotent Stem Cells. Stem cells international.

[155] Vethe H, Ghila L, Berle M, Hoareau L, Haaland OA, Scholz H (2019). The Effect of Wnt Pathway Modulators on Human iPSC-Derived Pancreatic Beta Cell Maturation. Front Endocrinol (Lausanne).

[156] Sharon N, Vanderhooft J, Straubhaar J, Mueller J, Chawla R, Zhou Q (2019). Wnt Signaling Separates the Progenitor and Endocrine Compartments during Pancreas Development. Cell reports.

[157] Peiris H, Bonder CS, Coates PT, Keating DJ, Jessup CF (2014). The beta-cell/EC axis: how do islet cells talk to each other?. Diabetes.

[158] Benninger RK, Piston DW (2014). Cellular communication and heterogeneity in pancreatic islet insulin secretion dynamics. Trends Endocrinol Metab.

[159] Peterson QP, Veres A, Chen L, Slama MQ, Kenty JHR, Hassoun S (2020). A method for the generation of human stem cell-derived alpha cells. Nature communications.

[160] Kodani N, Nakae J, Kobayashi M, Kikuchi O, Kitamura T, Itoh H (2020). FCoR-Foxo1 Axis Regulates alpha-Cell Mass through Repression of Arx Expression. iScience.

[161] Hartig SM, Cox AR (2020). Paracrine signaling in islet function and survival. J Mol Med (Berl).

[162] Ionescu-Tirgoviste C, Gagniuc PA, Gubceac E, Mardare L, Popescu I, Dima S (2015). A 3D map of the islet routes throughout the healthy human pancreas. Sci Rep.

[163] Cabrera O, Berman DM, Kenyon NS, Ricordi C, Berggren PO, Caicedo A (2006). The unique cytoarchitecture of human pancreatic islets has implications for islet cell function. Proc Natl Acad Sci U S A.

[164] Gregg BE, Moore PC, Demozay D, Hall BA, Li M, Husain A (2012). Formation of a human β-cell population within pancreatic islets is set early in life. J Clin Endocrinol Metab.

[165] Farhat B, Almelkar A, Ramachandran K, Williams SJ, Huang HH, Zamierowksi D (2013). Small human islets comprised of more β-cells with higher insulin content than large islets. Islets.

[166] Kojima N, Takeuchi S, Sakai Y (2014). Engineering of pseudoislets: effect on insulin secretion activity by cell number, cell population, and microchannel networks. Transplant Proc.

[167] Bosco D, Armanet M, Morel P, Niclauss N, Sgroi A, Muller YD (2010). Unique arrangement of alpha- and beta-cells in human islets of Langerhans. Diabetes.

[168] Lou YR, Leung AW (2018). Next generation organoids for biomedical research and applications. Biotechnol Adv.

[169] Montes-Olivas S, Marucci L, Homer M (2019). Mathematical Models of Organoid Cultures. Front Genet.

[170] Dahl-Jensen S, Grapin-Botton A (2017). The physics of organoids: a biophysical approach to understanding organogenesis. Development.

[171] Okuda S, Takata N, Hasegawa Y, Kawada M, Inoue Y, Adachi T (2018). Strain-triggered mechanical feedback in self-organizing optic-cup morphogenesis. Sci Adv.

[172] Berger E, Magliaro C, Paczia N, Monzel AS, Antony P, Linster CL (2018). Millifluidic culture improves human midbrain organoid vitality and differentiation. Lab Chip.

[173] Yan H, Konstorum A, Lowengrub JS (2018). Three-Dimensional Spatiotemporal Modeling of Colon Cancer Organoids Reveals that Multimodal Control of Stem Cell Self-Renewal is a Critical Determinant of Size and Shape in Early Stages of Tumor Growth. Bull Math Biol.

[174] Zbinden A, Urbanczyk M, Layland SL, Becker L, Marzi J, Bosch M (2021). Collagen and Endothelial Cell Coculture Improves β-Cell Functionality and Rescues Pancreatic Extracellular Matrix. Tissue Eng Part A.

[175] Brusko TM, Russ HA, Stabler CL (2021). Strategies for durable β cell replacement in type 1 diabetes. Science.

[176] Elizondo DM, Brandy NZD, da Silva RLL, de Moura TR, Ali J, Yang D (2020). Pancreatic islets seeded in a novel bioscaffold forms an organoid to rescue insulin production and reverse hyperglycemia in models of type 1 diabetes. Sci Rep.

[177] Nazeer MA, Karaoglu IC, Ozer O, Albayrak C, Kizilel S (2021). Neovascularization of engineered tissues for clinical translation: Where we are, where we should be?. APL Bioeng.

[178] Salg GA, Giese NA, Schenk M, Huttner FJ, Felix K, Probst P (2019). The emerging field of pancreatic tissue engineering: A systematic review and evidence map of scaffold materials and scaffolding techniques for insulin-secreting cells. J Tissue Eng.

[179] Mao D, Zhu M, Zhang X, Ma R, Yang X, Ke T (2017). A macroporous heparin-releasing silk fibroin scaffold improves islet transplantation outcome by promoting islet revascularisation and survival. Acta Biomater.

[180] Carlsson PO, Espes D, Sedigh A, Rotem A, Zimerman B, Grinberg H (2018). Transplantation of macroencapsulated human islets within the bioartificial pancreas betaAir to patients with type 1 diabetes mellitus. Am J Transplant.

[181] Siehler J, Blöchinger AK, Meier M, Lickert H (2021). Engineering islets from stem cells for advanced therapies of diabetes. Nat Rev Drug Discov.

[182] de Klerk E, Hebrok M (2021). Stem Cell-Based Clinical Trials for Diabetes Mellitus. Front Endocrinol (Lausanne).

[183] Ramzy A, Thompson DM, Ward-Hartstonge KA, Ivison S, Cook L, Garcia RV (2021). Implanted pluripotent stem-cell-derived pancreatic endoderm cells secrete glucose-responsive C-peptide in patients with type 1 diabetes. Cell Stem Cell.

[184] Shapiro AMJ, Thompson D, Donner TW, Bellin MD, Hsueh W, Pettus J Insulin expression and C-peptide in type 1 diabetes subjects implanted with stem cell-derived pancreatic endoderm cells in an encapsulation device. Cell Rep Med. 2021: 100466.

